# Effects of Alternative Offers of Screening Sigmoidoscopy and Colonoscopy on Utilization and Yield of Endoscopic Screening for Colorectal Neoplasms: Protocol of the DARIO Randomized Trial

**DOI:** 10.2196/17516

**Published:** 2020-08-05

**Authors:** Petra Schrotz-King, Michael Hoffmeister, Peter Sauer, Anja Schaible, Hermann Brenner

**Affiliations:** 1 Division of Preventive Oncology National Center for Tumor Diseases (NCT) German Cancer Research Center (DKFZ) Heidelberg Germany; 2 Division of Clinical Epidemiology and Aging Research German Cancer Research Center (DKFZ) Heidelberg Germany; 3 Interdisciplinary Endoscopy Center (IEZ) Heidelberg University Hospital Heidelberg Germany; 4 Department of Gastroenterology, Hepatology and Infectology Heidelberg University Hospital Heidelberg Germany; 5 Department of General, Visceral and Transplantation Surgery Heidelberg University Hospital Heidelberg Germany

**Keywords:** cross-sectional study, prospective randomized controlled two-arm intervention trial, endoscopy, screening colonoscopy, screening sigmoidoscopy, neoplasms, liquid and tissue biobank, human biosamples, early detection markers, blood, stool, urine, saliva, tissue, prevention and early detection program

## Abstract

**Background:**

Flexible sigmoidoscopy and colonoscopy are recommended screening options for colorectal cancer (CRC). Despite colonoscopy being offered for CRC screening in Germany, the uptake of this offer has been very limited.

**Objective:**

The objective of this study was to assess the potential for increasing use of endoscopic CRC screening and the detection of advanced colorectal neoplasms by offering the choice between use of flexible sigmoidoscopy and colonoscopy.

**Methods:**

The DARIO study includes a cross-sectional study (part I), followed by a prospective 2-arm randomized controlled intervention trial (part II) with an associated biobank study (part III). Participation is possible in part I of the DARIO study only, parts I and II, or all 3 study parts. After obtaining informed consent from the municipalities, 12,000 people, aged 50-54 years, from the Rhine-Neckar region in Germany were randomly selected from residential lists of the responsible population registries and invited to complete a standardized questionnaire to investigate the nature, frequency, timing, and results of previous CRC screening and eventual diagnostic colonoscopies. In study part II participants from study part I with no colonoscopy in the preceding 5 years are randomized into 2 arms: arm A offering screening colonoscopy only, and arm B offering both options, either screening colonoscopy or screening sigmoidoscopy. The primary endpoint is the proportion of participants in whom colorectal neoplasms >0.5 cm are detected and removed at screening endoscopy. The secondary endpoints are the detection rate of any neoplasm and use of any endoscopic screening. Part III of the study will use samples from participants in study part II to construct a liquid and tissue biobank for the evaluation of less invasive methods of early detection of colon cancer and for the more detailed characterization of the detected neoplasms. Blood, urine, stool, and saliva samples are taken before the endoscopy. Tissue samples are obtained from the neoplasms removed during endoscopy.

**Results:**

A total of 10,568 from 12,000 randomly selected women and men aged 50-54 years living in the Rhine-Neckar-Region of Germany have been invited for participation. The remaining 1432 (11.93%) could not be invited because they reached the age of 55 at the time of contact. Of those invited, 2785/10,568 (26.35%) participated in study part I; 53.60% (1493/2785) of these participants were female. Study parts II and III are ongoing.

**Conclusions:**

This study will answer the question if alternative offers of either screening sigmoidoscopy or screening colonoscopy will increase utilization and effectiveness of endoscopic CRC screening compared with an exclusive offer of screening colonoscopy. In addition, alternative noninvasive screening tests will be developed and validated.

**Trial Registration:**

German Clinical Trials Register DRKS00018932; https://www.drks.de/drks_web/navigate.do? navigationId=trial.HTML&TRIAL_ID=DRKS00018932

**International Registered Report Identifier (IRRID):**

DERR1-10.2196/17516

## Introduction

### Background

Colorectal cancer (CRC) is the third most common cancer and the second most common cause of cancer deaths globally, responsible for more than 1.8 million cases and more than 800,000 deaths annually [1]. In Germany there are annually approximately 60,000 cases and 25,000 deaths as a result of CRC [2]. Slow progression of the cancer through the adenoma–carcinoma sequence opens up promising possibilities for prevention and early detection interventions [3].

The majority of CRC cases could be prevented by detection and removal of adenomas at screening sigmoidoscopy or colonoscopy [4]. Several countries provide an opportunistic screening programme including colonoscopy (Austria, Czech Republic, Greece, Iceland, Luxembourg, Portugal, Slovakia); in very few countries organized screening programs offer either colonoscopy (Germany, Poland) or sigmoidoscopy (England and Italy). The United States and South Korea offer both endoscopic screenings, reporting uptake of up to 60% [5]. Screening colonoscopy has the advantage that adenomas can be detected and removed in the entire colon and rectum but the procedure requires full bowel cleansing starting the day before colonoscopy. Screening sigmoidoscopy detects adenomas only in the distal colon and rectum (where the majority of CRCs occur), but is less invasive and less demanding regarding bowel preparation, with an enema immediately prior to the procedure being sufficient. Thus, adherence to the offer of screening sigmoidoscopy is expected to be higher than adherence to the offer of screening colonoscopy.

Severe complications during or after colonoscopy or sigmoidoscopy are very rare [6]. Besides, participants greatly benefit from the removal of advanced adenomas that would develop into CRC in about 30% of the carriers within the next 10 years [7,8]; participants also benefit from removal of nonadvanced adenomas, which are also targets of screening endoscopy with a somewhat lower CRC transition rate [8-10].

Several large-scale randomized trials have demonstrated a major reduction of CRC incidence and mortality by screening sigmoidoscopy [11-18]. Even larger effects are expected from screening colonoscopy, but first results from the only large-scale randomized controlled trial assessing this question will not be available before mid-2020. Evidence from epidemiological studies suggests that the majority of CRCs and CRC deaths could be prevented by detection and removal of adenomas by screening colonoscopy [15,19], with somewhat lower effects but better adherence to be expected from screening sigmoidoscopy [15,19-21]. Overall, low adherence is the major limiting factor for a more effective prevention of CRC incidence and mortality within the population. In Germany, screening colonoscopy (but not screening sigmoidoscopy) has been offered for women and men aged 55 and older since the end of 2002. However, only 20%-25% of those eligible have utilized this screening offer within the first 10 years [10].

### Objectives

**Figure 1:**
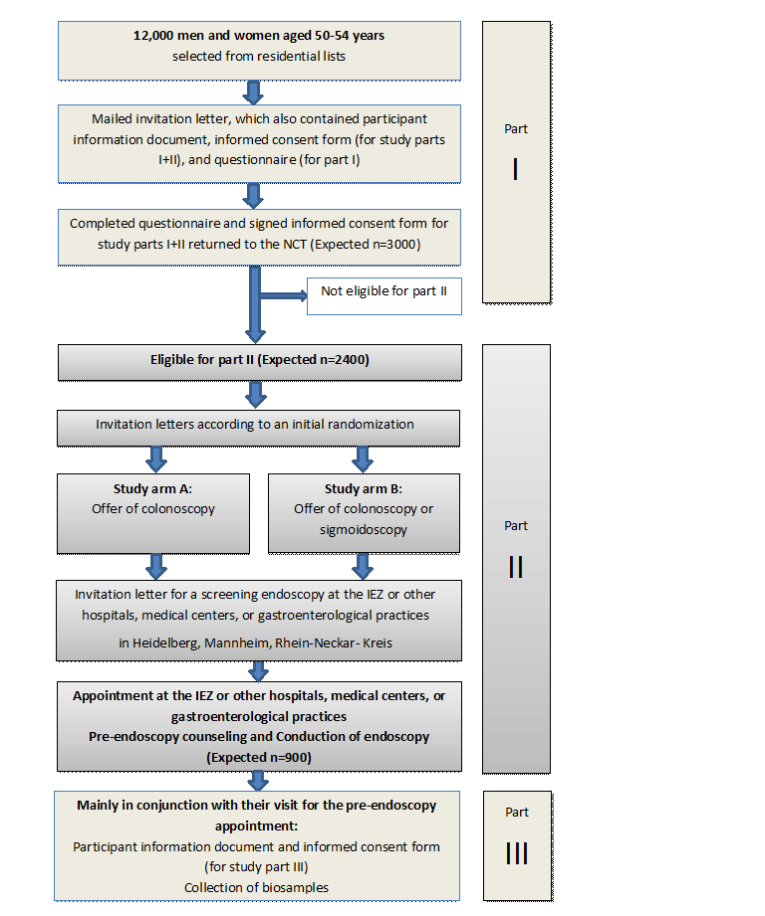
DARIO study design.

The DARIO (German title: “Darmkrebsprävention: Innovative Wege am Nationalen Centrum für Tumorerkrankungen [NCT]) study includes 3 study parts (Figure 1) and has 3 major objectives: part I of the study, an epidemiological cross-sectional study that assesses through a standardized questionnaire in a random sample of women and men aged 50-54 years type, frequency, date, and results of previous early detection examinations and of potential diagnostic colonoscopies for CRC.

Part II of the study, a randomized intervention study including eligible participants from part I, randomizes those into 2 arms: arm A, in which a free screening colonoscopy alone is offered, and arm B, in which one of the two endoscopic screening options is offered: a free screening colonoscopy or a free screening sigmoidoscopy. The main objective of part II is to assess if and by how much the complementary offer of the less invasive screening sigmoidoscopy will lead to a higher number of detected and removed neoplasms (>0.5 cm). Endoscopies are performed at the Interdisciplinary Endoscopy Center (IEZ) of the Heidelberg University Hospital according to common clinical practice. Tissue samples are obtained from the neoplasms removed during endoscopy and will be banked for diagnostics and research purposes at the NCT tissue bank.

Part III of the study builds up a liquid biobank from biosamples (blood, urine, stool, saliva) of study part II participants, with samples taken prior to endoscopy. The main objective of study part III is the evaluation of less invasive methods of CRC screening and further characterization of detected neoplasms and defining biomarkers or biomarker panels of different composition (such as proteins, metabolites, DNA, microRNA, methylation markers) obtained via different -omics platforms.

## Methods

### Study Design

The DARIO study is designed in 3 study parts: as a cross-sectional study (part I), followed by a prospective 2-arm randomized controlled intervention trial (part II) with an associated biobank study (part III) for biomarker evaluation as diagnostic tests of less invasive methods. The study design is shown in Figure 1. The trial was registered during recruitment of the participants in Deutsches Register Klinischer Studien on September 30, 2019.

### Study Part I: Cross-Sectional Screening for Previous Early Detection Examinations and Risk Factors

A total of 12,000 randomly selected potential participants aged 50-54 years living in the Rhine-Neckar region of Germany (main residence in Heidelberg, Mannheim, and Rhein-Neckar-Kreis, as defined by community codes) received a participant information document for study parts I + II and a short questionnaire by letter, and were invited by the DARIO study center at the National Center for Tumor Diseases in Heidelberg, Germany to participate in the study. Persons willing to participate were asked to sign and return the informed consent form for parts I + II of the study (included in the letter, together with a prepaid return envelope) and the questionnaire to the DARIO study center. The questionnaire includes questions on previous early detection examinations as well as questions concerning familial risk factors, lifestyle, health behavior, and nutritional factors.

Eligibility for parts II and III of the study is contingent on absence of a number of exclusion criteria that are specifically asked for in the questionnaire of part I. Exclusion criteria for parts II and III of the study are a history of CRC, a colonoscopy in the previous 5 years, a history of familial adenomatous polyposis or Lynch syndrome, and a severe illness which makes it impossible for the potential participant to visit the IEZ at the University Hospital Heidelberg or collaborating medical centers or gastroenterological practices in the Rhine-Neckar region (Heidelberg, Mannheim, and Rhein-Neckar-Kreis).

### Study Part II: Pre-Endoscopic Counseling and Endoscopic Screening in Study Arm A or B

Eligible participants are randomly allocated into either study arm A or B. Study arm A offers the participants a free screening colonoscopy, and in study arm B the eligible participants receive a letter, which offers the choice of either a free screening colonoscopy or a free screening sigmoidoscopy. The endoscopies take place at the IEZ or at other hospitals or medical centers or gastroenterological practices in the Rhine-Neckar region (Heidelberg, Mannheim, and Rhein-Neckar-Kreis).

To randomize the participants eligible for part II into arm A or arm B, a unique random number is generated for all 12,000 persons via a computer by a statistician with no involvement in the trial, before the persons are contacted in part I of the study, not knowing who will eventually participate in part I and who will be eligible for part II. Persons who agree to participate and who are eligible for part II are assigned into the respective study arm and a letter is sent to the participants inviting them to a pre-endoscopy counseling appointment at the NCT Study Center or at the IEZ. During pre-endoscopy consultation, the participants will receive routine patient information documents and, if they decide to have such an examination, they will sign the informed consent routinely used and required before endoscopy in clinical practice. Participants can be excluded from the study by the consulting physician due to underlying diseases or insufficient health status influencing endoscopy.

Screening colonoscopy and screening sigmoidoscopy are conducted at and under the responsibility of the performing center (hospitals, medical centers, or gastroenterological practices). Blinding of employees at the respective sites or at the study center is not feasible, because knowledge of endoscopy type(s) offered is a prerequisite for pre-endoscopy consultation and performing endoscopy. The study visit for the screening colonoscopy at the respective centers takes about 60 minutes for the screening colonoscopy and about 30 minutes for the screening sigmoidoscopy.

### Study Part III: Biosample Collection and Set up of a “Prevention Biorepository” for Biomarker Analysis

In conjunction with their visit for pre-endoscopy consultation at the NCT or IEZ, study participants willing to undergo endoscopy are informed in detail about study part III for which they are invited to donate biological samples (36 mL of blood, and samples of stool, urine, and saliva) for the evaluation of noninvasive or minimally invasive early detection markers. Biosamples are delivered directly to the Laboratory of the Division of Preventive Oncology at the NCT, the DARIO study center, without any delay. The stool sample is taken by the participants at their homes, collected prior to initiation of the large bowel preparation for colonoscopy, stored cool according to the manual, and delivered to the respective endoscopy center on the day of endoscopy. The sample reaches the NCT Laboratory on the same day and is processed and stored immediately. Exceptions occur with a maximum delay of 3 days for arrival or processing (eg, over weekends). The samples are then stored at 4°C for further processing or frozen directly at –80°C if no processing is required. All samples are processed according to the state-of-the-art standard operating procedures of the NCT Liquid Biobank, within 2-4 hours after taking the samples or entry of the biomaterial at the study center for the analysis of biomarkers. The samples are stored in barcoded vials and either frozen at –80°C or stored in liquid nitrogen for long-term storage. They are documented in a laboratory information management system (STARLIMS). For part III of the study, patients are asked to sign a separate specific informed consent form and a transfer agreement form for provision of biological samples.

### Data Collection and Documentation in the Coordinating Center

The unique participant number links biological samples, questionnaires, and endoscopy results. Information collected from endoscopy and histology reports is entered into a standardized study database by trained staff in the coordinating center at the NCT, using double data entry by 2 independent staff members. Data entries are checked through comparison of the corresponding data sets for inconsistencies and in case of differences in data sets, the data are validated by checking the original reports.

The information collected in the questionnaire in part I is documented by automated scanning of the questionnaires, optical verification of the scans by trained staff, and by applying comprehensive plausibility checks prior to statistical analysis. All collected information is stored at the coordinating center for at least 10 years after the end of the trial.

### Data and Biosample Analysis

Statistical analyses will be conducted using basic and advanced statistical methods for clinical epidemiological studies, including the analysis of biomarker data. The samples in the biobank built up in this trial will be used for determining the diagnostic value of novel biomarkers for early detection of colorectal neoplasms using standard receiver operating characteristic analyses for single markers and advanced biostatistics and bioinformatics tools for high-dimensional data obtained from -omics technologies.

The biospecimen will be used for identifying and evaluating biomarkers and biomarker signatures for cancer early detection and risk assessment. The most desirable highest scientific benefit from all biospecimen requires the determination of specific laboratory parameters and laboratory techniques according to the state of the art at the time of the analyses, which for most participants and most measurements will be years after recruitment. Examples of measurements anticipated at the time of conception of the DARIO study were measurements of defined metabolites in blood, stool, urine and saliva; circulating microRNA, especially in blood samples; single-nucleotide polymorphism analyses; next-generation sequencing; transcriptomics and application of several available and emerging -omics technologies, such as epigenomics, serolomics, proteomics, and stool metagenomics potentially related to the presence of early and advanced adenomas or colorectal carcinoma or both.

Long-term storage of the biosamples collected in this trial will enable timely validation of emerging promising early detection markers in the years to come.

### Method Against Bias

Data on confounding factors such as education, smoking, nutrition and diet, alcohol consumption, family history of cancer will be collected through the questionnaire in study part I. The trial is randomized to account for confounding factors. Extraction of clinical data from colonoscopy and pathology reports is accomplished in a blinded manner to avoid information bias. Data extraction and data entry (where applicable) are performed by 2 independent reviewers. Discrepant coding is resolved according to standard operating procedures to achieve the maximum accuracy possible. In addition, all recruiters and participants receive detailed instructions to ensure uniform collection and handling of biosamples. Preanalysis conditions, including sample transportation, are also documented to control for potential variation. All laboratory analyses are performed in a blinded fashion with respect to both treatment given and clinical/colonoscopy data.

### Sample Size Calculation

The current participation rate in the German screening colonoscopy program is 2%-3% per year among those eligible in the absence of personal invitations (performed as so-called opportunistic screening program). It has been repeatedly demonstrated (including own studies) that this participation rate can be at least doubled by personal invitation letters. Our estimates of recruitment numbers are rather conservative.

The sample size calculation is based on an anticipated participation rate of 25% (n=3000) in study part I out of 12,000 eligible participants and of 20% (n=2400) eligible participants for study part II, who are randomized into arm A or B (1200 participants in each arm).

In study arm A, 300/1200 participants (25.00%) are expected to undergo screening colonoscopy within 1 year, of whom 16.0% (48/300) are expected to have colorectal adenomas >0.5 cm detected and removed [22].

In study arm B, 240/1200 (20.00%) are expected to undergo screening colonoscopy and another 360/1200 (30.00%) are expected to undergo screening sigmoidoscopy within 1 year.

With an expected detection rate of neoplasms >0.5 cm of 16% and 12% by screening colonoscopy and screening sigmoidoscopy, respectively, the expected number of participants who have neoplasms >0.5 cm detected and removed is 81 (38 in arm A + 43 in arm B) [22,23].

The power to detect a significantly different rate of detection and removal of neoplasms >0.5 cm between both arms of study part II (48/1200 in arm A and 81/1200 in arm B) is 85% (two-sided chi-square test at α=.05).

As much as 90.0% (810/900) of participants undergoing endoscopy are expected to also participate in study part III. Evaluation of biomarker performance will be conducted according to standard methods of clinical epidemiology, bioinformatics, and biostatistics. Logistic regression models will be applied for individual biomarkers to construct prediction algorithm, and .632+ bootstrap [24] will be applied to adjust for potential overestimation of diagnostic performance. Areas under the receiver operating characteristic curves and their 95% confidence intervals, and sensitivity (true-positive rate) of each individual biomarker at cutoffs yielding 80% and 90% specificities (true‐negative rate) will be calculated.

In order to derive multimarker algorithms for the prediction of the presence of CRC, least absolute shrinkage and selection operator logistic regression models will be applied to markers that remain significant after multiple testing. The least absolute shrinkage and selection operator regression adapted to obtain models with the best prediction accuracy will be combined with .632+ bootstrap to adjust for overfitting. Prediction algorithms will be derived. Joint performance of biomarkers with known CRC risk factors (such as age, sex, family history, smoking, alcohol consumption, and dietary factors, information on which will be obtained from questionnaires) for predicting presence of neoplasms will additionally be evaluated in multivariable models. Analyses will be performed with statistical software R language and environment (version 3.5.3; R core team).

### Quality Assurance, Safety, and Benefit Risk Assessment

Ethical approval for the DARIO study was obtained from the Ethics Committee of the Medical Faculty of the University of Heidelberg (DARIO: S-686/2015). The study is registered at the German Registry for Clinical Studies (Deutsches Register Klinischer Studien, DRKS) with the DARIO-ID DRKS00018932 and at the StudyBox with the StudyBox Registry Number ST-D453 by OnkoZert of the German Cancer Aid (see Multimedia Appendix 1).

The screening endoscopies offered in the course of study part II are well-known routine procedures and established clinical practice [25]. They are recommended for CRC screening in the average risk population aged 50 or older by expert committees in multiple countries including Germany. Conduction of endoscopies (including informed consent, preparation, safety and quality considerations, insurance) follows routine clinical practice. The only difference in this study from clinical practice is that screening endoscopies otherwise not covered by health insurance in this age group in Germany (50-54 years) during the time of recruitment are offered free of charge to participants in the respective arms of study part II.

Advantages of both procedures to discover and remove preadenomatous, adenomatous, and cancerous lesions in the bowel are well known, as are their possible side effects. Both methods are valued as secure and controllable, with less risks and side effects to be expected with sigmoidoscopy due to the less demanding bowel cleansing, the shorter duration of the procedure, the smaller endoscopic intervention area in the bowel, and the reduced need for anesthetics. With adequate preparation, severe complications during or after colonoscopy or sigmoidoscopy are very rare and advantages in view of the screening prevail for both methods. Participants benefit from removal of advanced adenomas that would often develop into CRC and from removal of nonadvanced adenomas, which are also targets of screening endoscopy with a somewhat lower CRC transition rate.

The IEZ at Heidelberg University Hospital and the other hospitals or medical centers or gastroenterological practices in the Rhine-Neckar region routinely perform the procedures applied in this study within the course of diagnosis and treatment of colorectal tumors and precursors, on the basis of defined and quality-assured, internationally accepted standards and guidelines, as well as on the basis of local guidelines and standards. Patient briefing is conducted and informed consent is obtained following the clinical guidelines and practice of the hospital, medical center, or gastroenterologist practice. Participants are informed by a qualified physician on the endoscopic procedure(s) according to randomization (arm A, colonoscopy only; arm B, colonoscopy and sigmoidoscopy) and the potential anesthesia. Chances and risks are explicitly commented on during this conversation and participants have time to ask questions and to think about their decision regarding use of the offer of endoscopy. There are no disadvantages in case of denial at any time point within the course of the study. Potential light and severe complications occurring during endoscopy or during blood draw can be treated immediately at the Heidelberg University Hospital or the clinics close to the other medical centers or gastroenterologist practices.

The quality-controlled preanalytics with cold chain transport, time to freeze documentation, and barcoded processing and asservation of the collected biological samples are assured at the Preventive Oncology liquid biobank laboratories and the NCT tissue bank.

### Availability of Data and Materials

The future data set(s) supporting the conclusions of the trial will be made available upon reasonable request. Study materials, including study protocol, participant information documents, informed consents, questionnaire, the invitation letters to study part I and to study part II for arm A and for arm B as well as the letter of exclusion to study part II and the completed CONSORT checklist are provided as Multimedia Appendices 2–12.

### Ethics Approval and Consent to Participate

This study is conducted in accordance with the principles of the Declaration of Helsinki (of 1975, revised in 2000) and with the German laws/regulations. The study follows the good epidemiological practice guidelines and in the hospitals, medical centers, and practices the physician work according to good medical practice, a code of conduct and medical ethics for doctors.

Before the trial was initiated, the DARIO study protocol and any related document provided to the study participants were approved by the responsible Ethics Committee of the Medical Faculty of the University of Heidelberg (Federführende Ethikkommission) and the institutional Research Board (IRB or REB) (DARIO Study Number: S-686/2015). Before being admitted to the study, participants consent to participate after the nature, scope, and possible consequences of the study have been explained in understandable form (informed consent).

### Data Protection

This study is conducted in accordance with the General Data Protection Regulation (EU) 2016/679, the Data Safety Federal Protection Act, and the Data Protection Act of Baden-Württemberg. Study center is the Division of Preventive Oncology at the NCT of the German Cancer Research Center (GCRC/DKFZ) in Heidelberg. Documentation of programs and implemented databases is supervised within the legal frame and the IT and data protection rules of the DKFZ. The pre-endoscopy consultation and the colonoscopies are performed under the responsibility and based on the standard interview template and operating procedures of the IEZ of the Heidelberg University Hospital.

## Results

A total of 10,568 people from 12,000 randomly selected women and men aged 50-54 years living in the Rhine-Neckar-Region of Germany have been invited for participation of whom 22.99% (2430/10568) were at age 50, 22.99% (2430/10568) at age 51, 22.99% (2430/10568) at age 52, 15.99% (1690/10568) at age 53, and 15.00% (1585/10568) at age 54. Of those invited, 2785 (26.35%) participated in study part I, of which 53.60% (n=1493) were female. A total of 1432 (11.93%) people could not be invited because they reached the age of 55 at the time of contact and an additional pool of participants was not accessible at the time. Study parts II and III are ongoing. Of the 2781 study part I participants who completed the questionnaire, 1060 (38.12%) participants were screened by an endoscopy.

## Discussion

A large proportion of CRCs can be avoided by screening. Screening colonoscopy (but not screening sigmoidoscopy) has been offered in the German health care system for men and women at the age of 55 and older since the end of 2002, but participation rates have remained low. With this trial we aim to assess if and to what extent use of endoscopic screening can be increased by alternative offers of screening sigmoidoscopy and screening colonoscopy, and if and to what extent this increases detection rates of advanced colorectal neoplasms at the age between 50 and 54 years in the defined study population. Endoscopic screening at this age was so far not supported by the German health care system, and sigmoidoscopy is so far not offered for CRC screening at any age in Germany. The study is expected to create awareness among the population included in the study and their immediate family or friends who also get to know about the study and its purpose. The fact that people are invited to participate and get a cost-neutral colonoscopy or sigmoidoscopy may motivate people to undergo screening who would not have participated at all or at a later point in time. In addition, a biobank is set up that will allow development and validation of novel noninvasive or minimally invasive tests for risk assessment and confirming presence of colorectal neoplasms (eg, blood, stool and urine tests). The biobank with the potential biomarker analyses will be a gold mine for early detection research.

Limitations of the study also need to be acknowledged. The study population is only representing a small but representative area within Germany, namely, the Rhine-Neckar-Region, including the cities Heidelberg and Mannheim. The study can only include a certain manageable number of participants, who are provided endoscopic offers in addition to the already existing screening offers. Sample size will limit possibilities of in-depth analyses by subgroups. Long-term end points, such as CRC incidence and mortality, cannot be evaluated.

Despite its limitations, the study is expected to make a major contribution to develop more effective CRC screening strategies.

## Appendix

Multimedia Appendix 1 Accreditation Certificate for the DARIO Study from the German Cancer Society.

Multimedia Appendix 2 Study Protocol, DARIO (latest version).

Multimedia Appendix 3 Participant Information, DARIO Part I+II (latest version).

Multimedia Appendix 4 Participant Information, DARIO Part III (latest version).

Multimedia Appendix 5 Informed Consent, DARIO Part I+II (latest version).

Multimedia Appendix 6 Informed Consent, DARIO Part III (latest version).

Multimedia Appendix 7 DARIO Questionnaire.

Multimedia Appendix 8 Invitation Letter, DARIO Study Part I.

Multimedia Appendix 9 Invitation Letter, DARIO Study Part II A.

Multimedia Appendix 10 Invitation Letter, DARIO Study Part II B.

Multimedia Appendix 11 Exclusion Letter, DARIO Part II.

Multimedia Appendix 12 CONSORT 2010 Checklist.

## Abbreviations

CRC: colorectal cancer
NCT: Nationalen Centrum für Tumorerkrankungen
