# Supplementary material for: Effects of Alternative Offers of Screening Sigmoidoscopy and Colonoscopy on Utilization and Yield of Endoscopic Screening for Colorectal Neoplasms: Protocol of the DARIO Randomized Trial
Source: JMIR Res Protoc. 2020 Aug 5;9(8):e17516. doi: 10.2196/17516 (PMC7439136; doi:10.2196/17516)
Supplement: Multimedia Appendix 1 [file resprot_v9i8e17516_app1.pdf]

DKG KREBSGESELLSCHAFT

Akkreditierungsurkunde Studienboard

studybox

Die Studie

DARIO

SPONSOR: DKFZ/NCT Heidelberg,
Stiftung Deutsche Krebshilfe,
IEZ der Universitätsklinik Heidelberg

STUDIENLEITER: Herr Prof. Dr. med. H. Brenner

wurde vom Studienboard Darmkrebs der Deutschen Krebsgesellschaft positiv bewertet. Mit dieser Urkunde wird die Erfüllung der Kriterien für die Anerkennung einer Studie innerhalb des Zertifizierungssystems bescheinigt.

Registrier-Nr.:	ST-D453
Datum Akkreditierung:	22.11.2019
Gültigkeit Akkreditierung:	31.12.2022

Prof. Dr. med. Benz
Arbeitsgemeinschaft deutscher Darmkrebszentren (ADDZ)

Prof. Dr. med. Post
Zertifizierungskommission Darmkrebszentrum

Prof. Dr. med. Seufferlein
Zertifizierungskommission Darmkrebszentrum

Deutsche Krebsgesellschaft e.V.
Kuno-Fischer-Straße 8
14057 Berlin
Tel. (030) 322 93 29 0
E-Mail: service@krebsgesellschaft.de
www.krebsgesellschaft.de

Die aktuelle Liste der akkreditierten Studien ist unter www.studybox.de veröffentlicht.
