# Supplementary material for: Effects of Alternative Offers of Screening Sigmoidoscopy and Colonoscopy on Utilization and Yield of Endoscopic Screening for Colorectal Neoplasms: Protocol of the DARIO Randomized Trial
Source: JMIR Res Protoc. 2020 Aug 5;9(8):e17516. doi: 10.2196/17516 (PMC7439136; doi:10.2196/17516)
Supplement: Multimedia Appendix 2 [file resprot_v9i8e17516_app2.pdf]

NATIONALES CENTRUM FÜR TUMORERKRANKUNGEN HEIDELBERG

getragen von:
Deutsches Krebsforschungszentrum
Universitätsklinikum Heidelberg
Thoraxklinik-Heidelberg
Deutsche Krebshilfe

DARIO: Darmkrebsprävention – **I**nn**o**vative Wege am NCT

DARIO: Colorectal Cancer Prevention – Innovative Approaches at the NCT

Study Protocol

(Version: 1.3, Date: 09.08.2017)

Principal Investigator
Prof. Dr. Hermann Brenner, German Cancer Research Center

Division of Preventive Oncology at the NCT
Division of Clinical Epidemiology & Aging Research
German Cancer Research Center
Im Neuenheimer Feld 581, 69120 Heidelberg
e-mail: h.brenner@dkfz.de
Tel.: 06221-421300

Co-Investigator
Prof. Dr. Peter Sauer, Heidelberg University Clinic

Interdisciplinary Endoscopy Center
Heidelberg University Clinic
Im Neuenheimer Feld 410, 69120 Heidelberg
e-mail: peter.sauer@med.uni-heidelberg.de

UniversitätsKlinikum Heidelberg

Table of Contents

1. Summary 3
    1.1. Zusammenfassung (German) 3
    1.2. Summary (English) 3
2. Background and rationale 4
3. Study endpoints and objectives..7
4. Study design and study schedule..8
    4.1. Study design..8
    4.2. Study program 11
5. Inclusion and exclusion criteria 11
    5.1. Key inclusion criteria: 12
    5.2. Key exclusion criteria: 12
6. Study end..12
7. Safety considerations..12
    7.1. Screening endoscopy 12
    7.2. Blood draw..13
8. Statistical considerations..13
    8.1. Study, sample size and power calculation..13
    8.2. Endpoint analyses..14
    8.3. Confounding factors..14
    8.4. Biomarker analysis..14
9. Ethical and legal aspects 14
10. Responsibilities and data protection..15
    10.1. Study responsibilities 15
    10.2. Biomaterial management and documentation 16
    10.3. Data or material transfer to third parties 16
    10.4. Data analyses and presentations..16
11. Financial aspects 16
12. Voluntariness of and withdrawal from the study 16
13. Signatures..18

1. Summary

1.1. Zusammenfassung (German)

Mehrere epidemiologische Studien haben gezeigt, dass das Risiko kolorektaler Karzinome über einen Zeitraum von 10 Jahren nach endoskopischem Screening mit Entfernung präkanzeröser Veränderungen um bis zu 60-90% gesenkt werden kann. Diese Ergebnisse haben international zu unterschiedlichen Empfehlungen bezüglich des Einsatzes endoskopischer Screeningverfahren geführt, die bezüglich Art (Sigmoidoskopie versus Koloskopie) und Intervall der Screeninguntersuchungen variieren. Ein bevölkerungsweites Screening wird zumeist ab einem Alter von 50 Jahren empfohlen.

In Deutschland ist Darmkrebs die dritthäufigste Krebserkrankung, mit mehr als 60.000 Neuerkrankungsfällen und mehr als 25.000 Todesfällen pro Jahr. Die Mehrheit dieser Fälle könnte durch die frühzeitige Entdeckung und Entfernung von adenomatösen Veränderungen des Kolons oder Rektums während einer Screening-Koloskopie oder -Sigmoidoskopie verhindert werden. Die Screening-Koloskopie wird in Deutschland für Männer und Frauen ab einem Alter von 55 Jahren angeboten, jedoch bisher nur in geringem Maß genutzt (nur ca. 20 – 25% der teilnahmeberechtigten Personen haben in 2003-2012, den ersten 10 Jahren dieses Screeningangebots, an der Vorsorge-Koloskopie teilgenommen). Bei einem zunehmenden Anteil der älteren Bevölkerung in Deutschland wurde aber bereits einmal eine diagnostische Koloskopie durchgeführt, die einen vergleichbaren präventiven Effekt hat. Die Screening-Sigmoidoskopie wird in Deutschland, anders als in anderen Ländern, nicht angeboten.

Weltweit wird derzeit intensiv nach nicht oder nur minimal invasiven Alternativen für endoskopische Screening-Untersuchungen geforscht (z.B. Bluttests, Stuhltests, Urintests), deren Evaluation in einem Screening-Setting vordringlich ist. Diese bevölkerungsbezogen in der Rhein-Neckar-Region durchgeführte Studie verfolgt in drei Studienteilen daher drei wesentliche Ziele:

Studienteil I ist eine epidemiologische Querschnittstudie, in der mittels eines standardisierten postalischen Fragebogens Art, Häufigkeit, Zeitpunkt und Ergebnisse früherer Darmkrebs-Früherkennungsuntersuchungen und eventueller diagnostischer Koloskopien in einer Zufallsstichprobe 50-54-jähriger Männer und Frauen, gesamt und in Abhängigkeit des Risikofaktorenprofils für Darmkrebs untersucht werden sollen.

Studienteil II ist eine randomisierte Interventionsstudie, in der potentiell geeigneten und hieran interessierten Teilnehmern aus Studienteil I in zwei Gruppen randomisiert werden: Teilnehmer in Arm A wird eine kostenfreie Screening-Koloskopie angeboten, Teilnehmer in Arm B haben die Wahl zwischen einer kostenfreien Screening-Koloskopie oder Screening-Sigmoidoskopie. Mit diesem Studienteil soll untersucht werden, ob und in welchem Umfang die Erweiterung des Screening-Angebots um die weniger invasive Sigmoidoskopie zu einer höheren Inanspruchnahme endoskopischen Screenings und einer höheren Zahl entdeckter und entfernter Neoplasien führt (primärer Endpunkt: Zahl der entdeckten und entfernten kolorektalen Neoplasien >0,5 cm).

Ergänzend wird in **Studienteil III** mit Proben von Teilnehmern an Studienteil II eine Liquid- und Gewebe-Biobank zur Evaluation weniger invasiver Methoden der Darmkrebs-früherkennung und zur näheren Charakterisierung der entdeckten Neoplasien aufgebaut. Blut-, Urin-, Stuhl-, und Speichelproben werden vor Durchführung der Endoskopie, Gewebeproben werden aus den bei den Endoskopien entfernten Neoplasien gewonnen.

1.2. Summary (English)

Several epidemiological studies have shown that the risk for colorectal cancer can be reduced by 60 to 90 per cent by endoscopic screening with detection and removal of colorectal neoplasms. The results led to different recommendations and offers of endoscopic screening procedures in different countries, which vary with respect to type of endoscopic procedure

(sigmoidoscopy versus colonoscopy) and the time intervals of screening. Screening is commonly recommended from age 50 on in the average risk population.

In Germany, colorectal cancer (CRC) is the third most common cancer, with more than 60,000 new cases and more than 25,000 deaths per year. The majority of these cases could be prevented through early detection and removal of colorectal adenomas during screening colonoscopy or screening sigmoidoscopy. Screening colonoscopy is offered for colorectal cancer screening in Germany for men and women from the age of 55 years on. However participation rates are low (only 20 - 25% of all eligible persons participated in 2003-2012, the first 10 years of this screening offer). However, in an increasing proportion of the older German population diagnostic colonoscopies have been performed, which have a similarly strong preventive effect. In contrast to other countries, a screening sigmoidoscopy is not offered in Germany.

Worldwide, there is intensive research going on for the development of non-invasive or minimally invasive alternatives to endoscopic screening examinations (e.g. blood tests, stool tests, urine tests). Still, validation of such tests needs to be conducted in screening settings. Thus, this population-based study conducted in the Rhine-Neckar region is divided into three parts and has three major objectives

Part I of the study is an epidemiological cross-sectional study to assess type, frequency, date and results of previous early detection examinations to detect colorectal cancer and of potential diagnostic colonoscopies in a random sample of women and men from 50-54 years through a standardized questionnaire sent by mail. Analyses will be conducted for the total study population and according to risk factor profiles for colorectal cancer.

Part II of the study is a randomized intervention study including interested and potentially eligible participants from part I, who are randomized into two arms: participants in arm A are being offered a free screening colonoscopy, participants in arm B are offered one out of two endoscopic screening options: a free screening colonoscopy or a free screening sigmoidoscopy. In this part of the study, we will investigate if and by how much the extension of the screening offer by the less invasive screening sigmoidoscopy will lead to a higher number of detected and removed neoplasms (primary endpoint: number of detected and removed colorectal neoplasms >0.5 cm).

In addition, in **part III** of the study we will also set up a liquid and tissue biobank to be used for the evaluation of less invasive methods of colorectal cancer screening and further characterization of detected neoplasms. From participants of study part II, blood, urine, stool and saliva samples are collected prior to endoscopy, tissue samples will be produced from colorectal neoplasms removed during colonoscopy.

2. Background and rationale

Colorectal cancer (CRC) is the third most common cancer in Germany, accounting for more than 60,000 cases and more than 25,000 deaths every year. The majority of these cases could be prevented by detection and removal of adenomas at screening sigmoidoscopy or colonoscopy. Screening by sigmoidoscopy or colonoscopy is recommended by national expert panels in the average risk population from age 50 on, but neither of these screening examinations is offered to the average risk population below age 55 in the German health care system. Only screening colonoscopy is offered from age 55 on. Screening colonoscopy has the advantage that adenomas can be detected and removed in the entire colon and rectum, but it requires full bowel cleansing starting the day before the procedure. Screening sigmoidoscopy detects adenomas only in the distal colon and rectum (where the majority of CRCs occur), but is less invasive and less demanding regarding bowel preparation, an enema immediately prior to the procedure is sufficient. Thus, adherence to the offer of screening sigmoidoscopy is expected to be higher than adherence to screening colonoscopy.

Severe complications during or after colonoscopy or sigmoidoscopy are very rare. On the other hand, participants greatly benefit from removal of advanced adenomas that would develop into colorectal cancer in about 30% of the carriers within the next 10 years. Participants also benefit from removal of non-advanced adenomas, which are also targets of screening endoscopy with a somewhat lower colorectal cancer transition rate.

Several large-scale randomized trials have demonstrated major reduction of CRC incidence and mortality by screening sigmoidoscopy (1-4). Even larger effects are expected from screening colonoscopy, but first results from the only large scale RCT assessing this question will not be available before the mid of the 2020s. Evidence from epidemiological studies suggests that the majority of CRCs and CRC deaths could be prevented by detection and removal of adenomas by screening colonoscopy, with somewhat lower effects but better adherence to be expected from screening sigmoidoscopy (5, 6). Overall, low adherence is the major limiting factor for more effective prevention of CRC incidence and mortality within the population. For example, screening colonoscopy (but not screening sigmoidoscopy) has been offered in the German health care system for women and men aged 55 and older since the end of 2002. However, only 20-25% of those eligible have utilized this screening offer within the first 10-years (7). On the other hand, in an increasing proportion of the older German population diagnostic colonoscopies have been performed, i.e. colonoscopies that are conducted to follow-up on symptoms or positive fecal occult blood tests, which have a similarly strong preventive effect (6).

We hypothesize that alternative offers of either screening sigmoidoscopy or screening colonoscopy will increase utilization and effectiveness of endoscopic CRC screening compared to an exclusive offer of screening colonoscopy. We therefore propose a randomized trial in which participants are randomized into two arms, with arm A offering screening colonoscopy only, and arm B offering both options, either screening colonoscopy or screening sigmoidoscopy. The endoscopies will be performed at the Interdisciplinary Endoscopy Center (IEZ) of the Heidelberg University Hospital or at other hospitals or medical centers or gastroenterological practices in the Rhine-Neckar region (Heidelberg, Mannheim, Rhine-Neckar-Kreis) in the close vicinity to where the participants have their residence. The endoscopies will be performed according to common clinical practice. The IEZ, the practices or the NCT study physicians will inform the patient about the endoscopy in accordance to the appropriate patient information and informed consent..

Even higher participation rates might be achieved with minimally invasive or non-invasive screening tests, such as blood, urine or stool tests. We therefore will ask study participants to provide blood, urine, saliva and stool samples prior to the screening examination and we will use these samples to establish a biobank for the evaluation and identification of novel biomarkers for less invasive methods of colorectal cancer screening.

1. Atkin WS, Edwards R, Kralj-Hans I, Wooldrage K, Hart AR, Northover JMA, et al. Once-only flexible sigmoidoscopy screening in prevention of colorectal cancer: a multicentre randomised controlled trial Lancet 2010;375:1624-33.
2. Segnan N, Armaroli P, Bonelli L, Risio M, Sciallero S, Zappa M, et al. Once-only sigmoidoscopy in colorectal cancer screening: Follow-up findings of the Italian Randomized Controlled Trial – SCORE. J Natl Cancer Inst 2011;103:1310-22.
3. Schoen RE, Pinsky PF, Weissfeld JL, Yokochi LA, Church T, Laiyemo AO, et al. Colorectal-cancer incidence and mortality with screening flexible sigmoidoscopy. N Engl J Med 2012;366:2345-57.
4. Holme Ø, Løberg M, Kalager M, Bretthauer M, Hernan MA, Aas E, et al. Effect of flexible sigmoidoscopy screening on colorectal cancer incidence and mortality. JAMA 2014;312:606-15.
5. Brenner H, Stock C, Hoffmeister M. Effect of screening sigmoidoscopy and screening colonoscopy on colorectal cancer incidence and mortality: systematic review and meta-analysis of randomised controlled trials and observational studies. BMJ 2014;348:g2467.
6. Brenner H, Chang-Claude J, Jansen L, Knebel P, Stock C, Hoffmeister M. Reduced risk of colorectal cancer up to 10 years after screening, surveillance or diagnostic colonoscopy: Gastroenterology 2014;146:709-17.

7. Brenner H, Altenhofen L, Stock C, Hoffmeister M. Prevention, early detection, and overdiagnosis of colorectal cancer within 10 years of screening colonoscopy in Germany. Clin Gastroenterol Hepatol 2015;13:717-23.

3. Study endpoints and objectives

In this study, we will assess and compare, for the first time, utilization and relevant findings (defined as detected and removed colorectal neoplasms >0.5 cm; neoplasms include adenomas and cancer) of alternative offers of screening sigmoidoscopy and screening colonoscopy compared to the offer of screening colonoscopy only in the general population aged 50-54 years with no previous early detection examination for colorectal cancer.

The study, which is carried out in three parts, follows three major objectives:

Study part	Objective
I	To assess type, frequency, date and results of previous early detection examinations in women and men from 50-54 years.
II	To compare the utilization and effectiveness of two alternative endoscopic colorectal cancer screening offers, i.e. offer of free screening colonoscopy only or alternative offer of free colonoscopy or free screening sigmoidoscopy in the eligible population.
III	To build up and use a bio- and databank for the evaluation of biomarkers for colorectal cancer screening

The following specific endpoints will be evaluated:

Study part	Endpoint
I	Frequency of previous early detection examinations.
II	<u>Primary endpoint</u>:<br>– Proportion of participants in whom colorectal neoplasms > 0.5 cm that are detected and removed at screening endoscopy.<br><br><u>Secondary endpoints</u>:<br>– Utilization rate of any screening endoscopy.<br>– Detection rate of any neoplasm.<br>– Patient preference with respect to type of screening endoscopy (proportion).<br>– Safety of the applied procedures, complication rates (established routine procedures, standard monitoring of complications).
III	Sensitivity, specificity, positive and negative predictive values, area under the curve (AUC) of diagnostic tests for detection of CRC and colorectal adenomas.

4. Study design and study schedule

4.1. Study design

The study is planned as a cross-sectional study (Part I), followed by a prospective two-arm randomized controlled intervention trial (Part II) with an associated biobank study (Part III). Participation is possible in part I only, parts I and II, or all three parts.

Part I: 12,000 people, aged 50-54 years, from the Rhine-Neckar region (Heidelberg, Mannheim, Rhine-Neckar-Kreis) will be randomly selected from residential lists of the responsible population registries. To obtain these data, all municipalities in the study region are first contacted and asked for their consent to obtain a random population sample through the regional data center. After consent is obtained, the data center will draw a random sample based on the eligibility criteria (age 50-54 years, residency in Heidelberg, Mannheim or Rhine-Neckar Kreis). Data transferred to the study center at the German Cancer Research Center include name, address, gender and date of birth of each inhabitant. The transfer of the data from the registry offices to the German Cancer Research Center is justified by §29 Abs. 1 of the "Meldegesetz für Baden-Württemberg" (http://www.landesrecht-bw.de) for approved population samples drawn for scientific use.

The randomly drawn persons will receive a participant information for study parts I+II and a short questionnaire by letter, and will be invited to participate in the study. Persons willing to participate are asked to sign and return the informed consent for parts I+II of the study (also included in the letter) and the questionnaire to the DARIO study center at the NCT. The questionnaire includes questions on previous early detection examinations, questions concerning familial risk factors, lifestyle, health behavior and nutritional factors (completion will take about 15 minutes). Eligibility for part II of the study is contingent on absence of a number of exclusion criteria outlined in section 5.2 and specifically asked for in the questionnaire of part I. Participants not eligible for part II will be informed so, with explanations of the reasons, by the study center by mail.

Part II: Eligible participants will be randomly allocated into either study arm A or B:

- Arm A: Offer of free screening colonoscopy at the IEZ or at other hospitals or medical centers or gastroenterological practices in the Rhine-Neckar region (Heidelberg, Mannheim, Rhine-Neckar-Kreis)
- Arm B: Offer of free screening colonoscopy or free screening sigmoidoscopy at the IEZ or at other hospitals or medical centers or gastroenterological practices in the Rhine-Neckar region (Heidelberg, Mannheim, Rhine-Neckar-Kreis)

In order to randomize the persons eligible for part II into arm A or arm B, a unique random number will be generated for all 12,000 persons before they are contacted in part I of the study, not knowing who will eventually participate in part I and who will be eligible for part II. Using a simple randomization approach, the list will then be sorted according to the random number: the first 6,000 will be assigned into arm A, the second 6,000 will be assigned into arm B. Persons who agree to participate and who are eligible for part II will then be assigned into the study arm that was pre-selected randomly before the start of the study. In larger trials such as this one, simple randomization can be trusted to achieve similar numbers in the study arms at the end of the study. After randomization, a letter will be sent to the participants inviting them to a pre-endoscopy counseling appointment at the IEZ or at another hospital or medical center or gastroenterological practice in the Rhine-Neckar region (Heidelberg, Mannheim, Rhine-Neckar-Kreis). During pre-endoscopy consultation, the participants will receive routine patient information for persons undergoing colorectal endoscopy according to their study arm and, if they decide to have such an examination, they will sign the informed consent routinely used and required before endoscopy in clinical practice.

Screening colonoscopy and screening sigmoidoscopy will be conducted at and under the responsibility of the IEZ of the Heidelberg University Hospital or the other hospitals or medical

centers or gastroenterological practices in the Rhine-Neckar region (Heidelberg, Mannheim, Rhine-Neckar-Kreis). Blinding of employees at these sites or at the NCT is not required and not feasible, because knowledge of endoscopy type(s) offered is a prerequisite for pre-endoscopy consultation and conduction of endoscopy.

Part III: In conjunction with their visit for pre-endoscopy consultation at the IEZ or at other hospitals or medical centers or gastroenterological practices in the Rhine-Neckar region (Heidelberg, Mannheim, Rhine-Neckar-Kreis) study participants willing to undergo endoscopy will be informed in detail on study part III in which they are invited to donate biological samples (36 ml of blood, and samples of stool, urine, and saliva) for the evaluation of non-invasive or minimally invasive early detection markers. For this third part of the study, patients will be asked to sign a separate specific informed consent and a transfer agreement for provision of biological samples. The study visit for the screening colonoscopy at the IEZ or at other hospitals or medical centers or gastroenterological practices in the Rhine-Neckar region (Heidelberg, Mannheim, Rhine-Neckar-Kreis) will take about 60 minutes for the screening colonoscopy and about 30 minutes for the screening sigmoidoscopy.

The flow chart below (Figure 1) illustrates the study design:

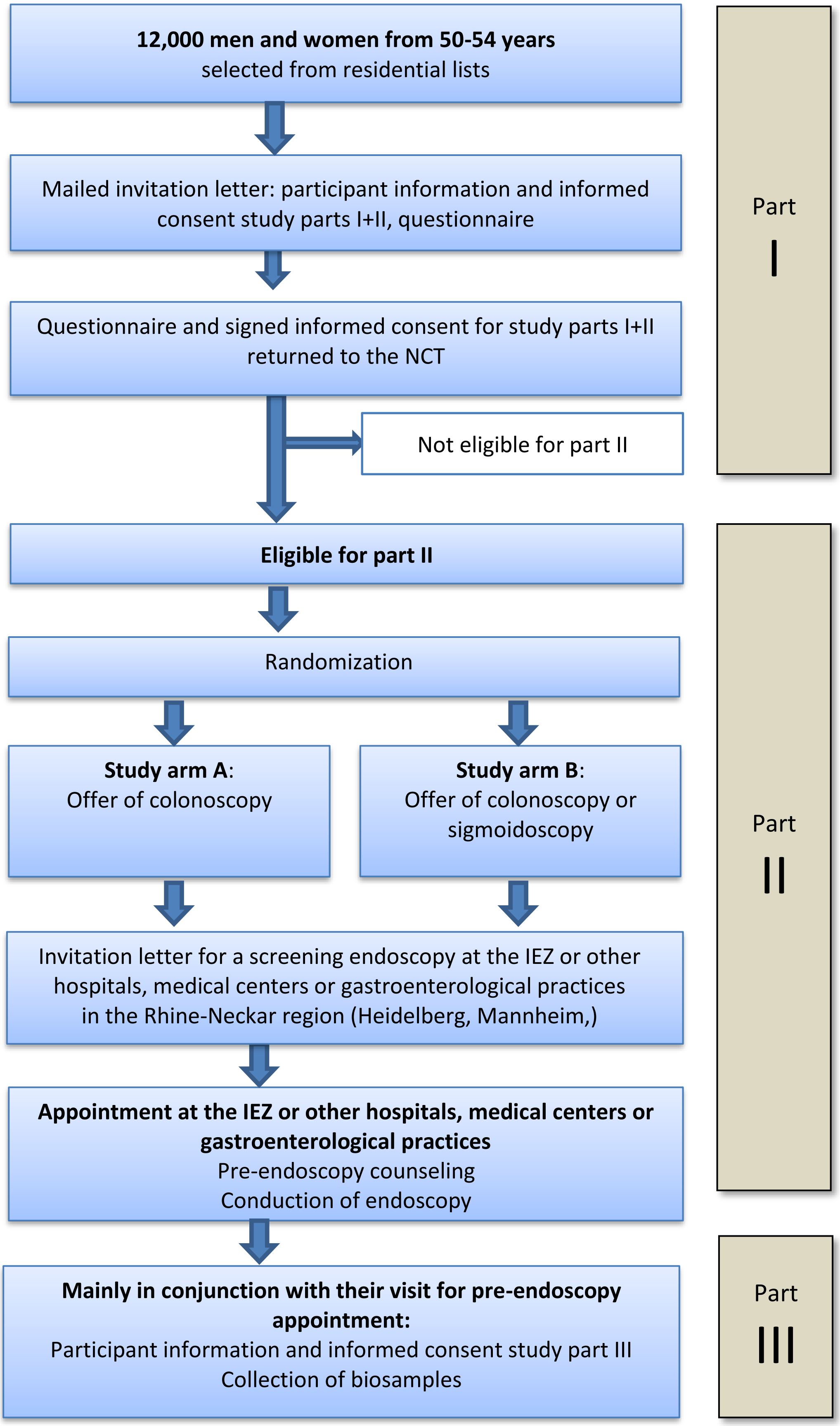

Figure 1. Overall study concept.

4.2. Study program

1. The NCT study center will send invitation letters to 12,000 randomly selected persons containing general information concerning the study, a detailed participant information form, a questionnaire, and an informed consent sheet for study parts I and II. Up to two reminders will be sent to non-responders after several weeks (up to 3 mailings in total).
2. Participants interested to take part in the study are asked to sign the inform consent for part I and part II and return the completed questionnaire to the NCT study center. At the NCT study center, inclusion and exclusion criteria for participation in study part II will be checked by the study personnel and be documented.

If all inclusion criteria are fulfilled and no exclusion criterion is met the participants willing to take part in part II will be randomized into study arm A or B, and will be invited by letter to schedule an appointment with the study center for pre-endoscopy counseling at the IEZ of the Heidelberg University Hospital or at other hospitals or medical centers or gastroenterological practices in the Rhine-Neckar region (Heidelberg, Mannheim, Rhine-Neckar-Kreis) in the close vicinity to where the participants have their residence. Non-responders will be reminded after several weeks by mail or phone call. If eligibility criteria are not met for study part II, participants will be sent a letter explaining why no screening endoscopy is offered.

3. At the IEZ of the Heidelberg University Hospital or at other hospitals or medical centers or gastroenterological practices in the Rhine-Neckar region (Heidelberg, Mannheim, Rhine-Neckar-Kreis) in the close vicinity to where the participants have their residence specific information on colonoscopy (Arm A) or colonoscopy and sigmoidoscopy (Arm B) is given during pre-endoscopy counseling.
4. In case the participant gives written informed consent: scheduling and conduction of colonoscopy or sigmoidoscopy (according to study arm) at the IEZ or at other hospitals or medical centers or gastroenterological practices in the Rhine-Neckar region (Heidelberg, Mannheim, Rhine-Neckar-Kreis) in the close vicinity to where the participants have their residence.
6. At the pre-endoscopy counseling, study participants will receive further detailed information on study part III by trained study personnel, and, upon written informed consent, they will be requested to donate blood (maximum 36 ml), urine and saliva samples. Furthermore, a stool kit, including an immunochemical fecal occult blood test and information about the application and a stool collection kit will be handed out to the participants for use at home. Participants are requested to hand over the stool sample at the endoscopy visit at the IEZ or at other hospitals or medical centers or gastroenterological practices in the Rhine-Neckar region (Heidelberg, Mannheim, Rhine-Neckar-Kreis) in the close vicinity to where the participants have their residence (from where it will be transferred to the NCT) or to send it via mail or courier service to the NCT (material for returning stool samples by mail will be provided). All biosamples will be stored in a biobank within the Division of Preventive Oncology at the NCT. Biomarker analyses will be carried out at NCT or national or international collaboration partners.
7. If polyps were removed during endoscopy, a tissue sample will be requested after pathology examination and transferred for inclusion into the biobank of the DARIO study.

5. Inclusion and exclusion criteria

The study population is randomly selected from residential lists of the population registries of the study region, ensuring maximum possible representativeness and generalizability.

5.1. Key inclusion criteria:

Inclusion criteria for this study:

- Women or men
- Age 50 (>49) to 54 (<55) at the day of invitation to the study
- Main residence in Heidelberg, Mannheim and Rhine-Neckar-Kreis (as defined by community codes)
- Ability to speak and understand German language
- Ability to give legally binding informed consent

5.2. Key exclusion criteria:

Participants will not be eligible for part II and part III of the study if they

- have a history of colorectal cancer.
- have had a colonoscopy in the previous 5 years.
- have a history of FAP (Familial Adenomatous Polyposis) or Lynch Syndrome.
- are severely ill and not able to visit the IEZ or other hospitals or medical centers or gastroenterological practices in the Rhine-Neckar region (Heidelberg, Mannheim, Rhine-Neckar-Kreis).

6. Study end

The study duration for the initial recruitment phase is 3 years, but prolongation of recruitment is envisaged, depending on participation and financial resources.

7. Safety considerations

7.1. Screening endoscopy

The screening endoscopies offered in the course of study part II are well known routine procedures and established clinical practice for decades. They are recommended for colorectal cancer screening in the average risk population aged 50 or older by expert committees in multiple countries including Germany. Conduction of endoscopies (including informed consent, preparation, safety and quality considerations, insurance) will follow routine clinical practice. The only difference from clinical practice is that screening endoscopies otherwise not covered by health insurance in this age group (50-54) will be offered free of charge to participants in the respective arms of study part II.

Advantages of both procedures to discover and remove pre-adenomatous, adenomatous and cancerous lesions in the bowel are well known, just as possible side effects. Both methods are valued as secure and controllable, with less risks and side-effects to be expected with sigmoidoscopy due to the less demanding bowel cleansing, the shorter duration of the procedure, the smaller endoscopic intervention area in the bowel, and the reduced need for anesthetics. With adequate preparation severe complications during or after colonoscopy or sigmoidoscopy are very rare and advantages in view of the screening prevail for both methods. Participants greatly benefit from removal of advanced adenomas that would often develop into colorectal cancer. Participants also benefit from removal of non-advanced adenomas, which are also targets of screening endoscopy with a somewhat lower colorectal cancer transition rate.

The IEZ at Heidelberg University Hospital and the other hospitals or medical centers or gastroenterological practices in the Rhine-Neckar region (Heidelberg, Mannheim, Rhine-Neckar-Kreis) routinely perform the procedures applied in this study within the course of diagnosis and treatment of colorectal tumors and precursors, on the basis of defined and

quality-assured, internationally accepted standards and guidelines, as well as on the basis of local guidelines and standards.. Patient briefing and informed consent are conducted following the clinical guidelines and practice of the hospital, medical center or gastroenterologist practice. In particular, participants are informed by a qualified physician on the endoscopic procedure(s) according to randomization (arm A: colonoscopy, arm B: colonoscopy and sigmoidoscopy) and the potential anesthesia. Chances and risks will be explicitly commented on during this conversation and participants will have adequate time to ask questions and to think about their decision regarding use of the offer of endoscopy. There will be no disadvantages in case of denial at any time point within the course of the study. Potential light and severe complications occurring during endoscopy or during blood draw can be treated immediately at the Heidelberg University Hospital or the clinics close to the other medical centers or gastroenterologist practices. Indemnity insurance in the unlikely case of harms caused by sigmoidoscopy or colonoscopy is covered by the University Clinics or the corresponding performing clinics, or medical centers or the gastroenterologist's insurances.

7.2. Blood draw

In study part III, a blood sample (maximum 36 ml) for evaluating potential blood markers for early detection of colorectal cancer will be drawn. Whenever possible, this will be implemented along with a blood draw routinely done at the pre-endoscopy visit to avoid additional venipuncture. In any case, participants will be fully informed on the small risks of venipuncture (see also participant information for part III of the study) at the blood draw, which will be done according to the highest possible standards by qualified study personnel.

8. Statistical considerations

8.1. Study, sample size and power calculation

The current participation rate in the German screening colonoscopy program is 2-3% per year among those eligible in the absence of personal invitations (performed as so called opportunistic screening program). It has been repeatedly demonstrated (including own studies) that this participation rate can be at least doubled by personal invitation letters. In sigmoidoscopy trials in other countries, much higher participation rates up to 50% and higher have been achieved (references 1-4 in section 2.1). Thus, our estimates of recruitment numbers are rather conservative:

In total, 12,000 people aged 50-54 will be approached by letter, with up to two reminder letters. We anticipate a participation rate of 25% in study part I (n=3000) and of 20% eligible persons in study part II (n=2400), who will be randomized into Arm A or Arm B (1200 participants in each arm).

Arm A: 300 participants in study arm A (25%) are expected to undergo screening colonoscopy within one year, of whom 16% (n=48) are expected to have colorectal adenomas >0.5 cm detected and removed.

Arm B: 240 (20%) are expected to undergo screening colonoscopy and another 360 (30%) are expected to undergo screening sigmoidoscopy in study arm B within one year. With an expected detection rate of neoplasms >0.5 cm of 16% and 12% by screening colonoscopy and screening sigmoidoscopy, respectively, the expected number of participants who have neoplasms >0.5 cm detected and removed is 38 + 43 = 81.

The power to detect a significantly different rate of detection and removal of neoplasms >0.5 cm between both arms of study part II (48/1200 in arm A and 81/1200 in arm B is 85% (two-sided Chi-Square Test at alpha=0.05).

90% of participants undergoing endoscopy are expected to also participate in study part III (n=864). Evaluation of biomarker performance will be conducted according to standard methods of clinical epidemiology, bioinformatics and biostatistics (see also section 9.4).

8.2. Endpoint analyses

The primary analysis of study part II described here will be an intention-to-screen analysis with the primary endpoint detection of any neoplasm >0.5 cm. For secondary endpoints see section 3.

Primary analyses will include both, men and women. Additional analyses will be conducted stratified according to sex and other factors, for example lifestyle- and health-related ones.

8.3. Confounding factors

Data on confounding factors like education, smoking, nutrition and diet, alcohol consumption, family history of cancer etc. will be collected through the questionnaire in study part I.

8.4. Biomarker analysis

The biospecimen (study part III) will be used for identifying and evaluating biomarkers and biomarker signatures for cancer early detection and risk assessment. Knowledge about biomarkers and technologies assessing biomarkers that are potentially relevant for cancer early detection and risk assessment are developing very fast. It is therefore neither possible nor meaningful to define a definitive list of biomarkers to be measured a priori. The most desirable highest scientific benefit from all biospecimens rather requires the determination of specific laboratory parameters and laboratory techniques according to the state-of-the-art at the time of the analyses, which for most participants and most measurements will be years after recruitment. Examples of measurements anticipated at this time include measurements of defined metabolites in blood, stool, urine and saliva, circulating RNA and DNA especially in blood samples, SNP analyses and application of several available and emerging –omics technologies, such as genomics, epigenomics, serolomics, proteomics, metabolomics, stool metagenomics, next generation sequencing and transcriptomics of tumor tissue.

Statistical analyses will be conducted using the basic and advanced statistical methods for clinical epidemiological studies, including the analysis of biomarker data. The samples in the biobank built up in this trial will be used for determining the diagnostic value of novel biomarkers for early detection of colorectal neoplasms using standard ROC analyses for single markers and advanced biostatistics and bioinformatics tools for high dimensional data obtained from –omics technologies.

The analyses will be performed by qualified epidemiologists, biostatisticians or bioinformaticians of the Division or at DKFZ, or in cooperation with qualified external partners.

9. Ethical and legal aspects

This study will be conducted in accordance with the principles of the "Declaration of Helsinki" and with the German laws/regulations. The study will follow the GEP (good epidemiological practice) guidelines, in the hospitals, medical centers and practices the physician will work according to good medical practice, a code of conduct and medical ethics for doctors.

The principal investigator will submit the protocol and any related document provided to the participant to an Independent Ethics Committee (IEC). Before being admitted to the study, the

participant must consent to participate after the nature, scope and possible consequences of the study have been explained in understandable form.

10. Responsibilities and data protection

10.1. Study responsibilities

This study will be conducted in accordance with the Federal Data Protection Act.

Study center is the Division of Preventive Oncology at the National Center for Tumor Diseases (NCT) of the German Cancer Research Center (DKFZ) in Heidelberg headed by the principal investigator, Prof. Dr. med. Hermann Brenner. Under the responsibility of Prof. Dr. med. Hermann Brenner all processes of participant recruitment, study information, informed consent to the study, prevention counseling, data and biosample collection, data input, storage and documentation are coordinated. Documentation of programs and implemented databases is supervised within the legal frame and data protection rules of the DKFZ.

Under the responsibility of Prof. Dr. med Peter Sauer (Heidelberg University Hospital) the pre-endoscopy consultation and the endoscopies of both study arms are performed, based on the standard interview template and operating procedures of the IEZ of the Heidelberg University Hospital.

Under the responsibility of the defined gastroenterologist in other hospitals or medical centers or gastroenterological practices in the Rhine-Neckar region (Heidelberg, Mannheim, Rhine-Neckar-Kreis) the pre-endoscopy consultation and the endoscopies of both study arms are performed, based on the standard interview template and operating procedures of the corresponding hospital, medical center or gastroenterological practice.

The quality-controlled processing, asservation, documentation and analysis of novel biomarkers of the collected biological samples are assured at the Preventive Oncology laboratories and liquid biobank and the NCT tissue and liquid biobank.

All persons working in the study have to sign a confidentiality agreement and are committed to protect the confidentiality in view of study participants and of all collected data and samples.

For the identification of participants, biosamples and any study participant related data, each participant receives a unique personal identification number (Participant ID). At the earliest possible time, personal identifying data (name, address, informed consent form) will be separated from any participant related additional clinical, analytical or questionnaire data and stored separately in a separate database with access strictly restricted to named study personnel in charge of participants contacts.

The names of the study participants and all other confidential information are subject to medical confidentiality and the provisions of the Data Protection Act of Baden-Württemberg.

Data acquisition, management and documentation are kept at the DKFZ and are conducted according to established standard operating procedures.

Only pseudonymized data will be used for analysis. The key for identification of participants will solely and only be held at the DKFZ. Third parties will not have access to the original study data. Pseudonomized data and samples transferred to cooperation partners before anonymization will contain a participant ID other than the main participant ID used at DKFZ.

Personal identifying data of participants will be held up to completion of all necessary data (maximum 15 months from recruitment) and deleted thereafter. From that time point data and samples will be stored anonymously. After 30 years, DKFZ will re-assess whether anonymized data and samples will still be needed, or whether data should be deleted and samples should be destroyed.

10.2. Biomaterial management and documentation

The biomaterial collected (blood, urine, stool, saliva) will be processed, stored and analyzed at DKFZ pseudonymously, labeled only with an identification number. Endoscopic biopsy material will be stored at the NCT tissue bank in Heidelberg, at the Institute of Pathology of the Heidelberg University Hospital. Specific analyses of the materials collected are solely conducted according to the informed consent of the participant, in appropriate labs with approved standard operation procedures, either at the DKFZ or externally, possibly at remote locations, but exclusively with permission of the principal investigator.

Corresponding clinical data e.g. results of the colonoscopy will be stored in addition to the biosamples. They are needed and will be used for evaluating diagnostic performance of the biomarkers (e,g. by comparing biomarkers with findings at colonoscopy). The investigators, their group members, as well as cooperation partners can get access to pseudonymized biosamples and corresponding clinical data for biomarker evaluations that are in line with the study protocol.upon request to the PI by a short written proposal. Study participants have the right to explicitly demand destruction of their biomaterial in case of withdrawal from the study. If study biomaterial already has been anonymized (after the deletion of personal identifying data of participants) deletion of biomaterial will not be possible or feasible anymore.

10.3. Data or material transfer to third parties

Data or biomaterial transferred to a third party will be de-identified, that means anonymized to that third party. The key for identification of participants will solely and only be held at the DKFZ.

10.4. Data analyses and presentations

Statistical and epidemiological analyses and outcomes will solely be performed with pseudonymized data of the study in aggregated form. Thus, conclusions on single persons or data of single persons will not be possible. Study data will only be used in the framework of the described project and the study objectives. Research results will only be published for scientific reasons.

11. Financial aspects

The study will be financed via the NCT 3.0 funding. There is no external sponsor. Neither the investigator nor any other study staff has financial or other conflicting interests in the outcome of the study.

12. Voluntariness of and withdrawal from the study

It will be emphasized that participation is voluntary and that no disadvantages or adverse consequences will arise due to refusal. The participant will only be included into the study if he/she consents in written form. At any time prior to anonymization, the study participant will have the right to withdraw without having to give any explanation and without any disadvantage. The withdrawal will be either in written form or verbal (in this case a note will be written by the study staff).

After withdrawal, the participant will not be contacted anymore by the study center. However, “passive” completion of data (e.g. via the hospital information system or treating physicians)

may still be conducted if permitted by the participant. Already collected data and samples will be used and analyzed further unless the participant objects.

Also, the participant can request the deletion of his personal identifying data (name, address) and samples at any point of time prior to anonymization. Complete withdrawal including deletion of all by then collected data and/or samples must be requested in written form (or by telephone with subsequent written confirmation). After complete anonymization, data and samples will be stored anonymously for an unlimited time. Unlimited storage of samples and data will enable the use of novel specific technology in the future, considering further development in scientific practice and knowledge.

13. Signatures

The present protocol was subject to critical review and has been approved in the present version by the person who signed the document. The information contained is consistent with the Declaration of Helsinki and Good Epidemiologic Practice.

Date: 09.08.2017

Signature of principle investigator:

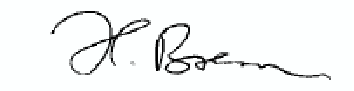

____

Prof. Dr. med. Hermann Brenner
Head, Division of Clinical Epidemiology and Aging Research
and Division of Preventive Oncology
German Cancer Research Center
