# Supplementary material for: Effects of Alternative Offers of Screening Sigmoidoscopy and Colonoscopy on Utilization and Yield of Endoscopic Screening for Colorectal Neoplasms: Protocol of the DARIO Randomized Trial
Source: JMIR Res Protoc. 2020 Aug 5;9(8):e17516. doi: 10.2196/17516 (PMC7439136; doi:10.2196/17516)
Supplement: Multimedia Appendix 3 [file resprot_v9i8e17516_app3.pdf]

Abteilung Präventive Onkologie
Nationales Centrum für
Tumorerkrankungen (NCT)
Im Neuenheimer Feld 460
69120 Heidelberg
DARIO Studienzentrum:
Telefon: 06221 - 56-34322

NATIONALES CENTRUM
FÜR TUMORERKRANKUNGEN
HEIDELBERG

getragen von:
Deutsches Krebsforschungszentrum
Universitätsklinikum Heidelberg
Thoraxklinik-Heidelberg
Deutsche Krebshilfe

DARIO: **Dar**mkrebsprävention – **I**nn**o**vative Wege am NCT

Teilnehmerinformation, Studienteile I und II

Sehr geehrte Damen und Herren,

Darmkrebs ist eine Krebserkrankung, die sich durch eine rechtzeitige Vorsorge meist verhindern lässt. Nationale und internationale Expertenvereinigungen empfehlen, bereits ab dem Alter von 50 Jahren mit der Darmkrebsvorsorge zu beginnen. Eine sogenannte präventive Darmspiegelung (Koloskopie) wird von den gesetzlichen Krankenkassen in Deutschland jedoch erst ab 55 Jahren angeboten. Mit der DARIO Studie möchten wir untersuchen, welcher zusätzliche Nutzen durch das Angebot einer Darmspiegelung bereits im Alter von 50-54 Jahren erreicht werden könnte.

Wir möchten Sie daher herzlich einladen, an der DARIO-Studie teilzunehmen und Sie bitten, einen leicht zu beantwortenden Fragebogen auszufüllen (Studienteil I). Falls für Sie eine präventive Darmspiegelung in Frage kommt, werden wir Ihnen anbieten, wie von Experten empfohlen, eine solche bereits vor dem 55. Geburtstag durchführen zu lassen (Studienteil II).

Ihre Adressdaten wurden uns vom Einwohnermeldeamt für diese wissenschaftliche Umfrage zur Verfügung gestellt.

Studienablauf

Wir möchten Sie gerne bitten, den beiliegenden kurzen Fragebogen auszufüllen. Sie werden dafür ca. 15 Minuten benötigen. Sollten Sie Fragen zur Teilnahme oder zum Ablauf haben, rufen Sie uns gerne an (Telefonnummer siehe oben). Für die Teilnahme an der Studie benötigen wir Ihre schriftliche Einwilligung auf der beiliegenden Einverständniserklärung.

Per Post werden wir Ihnen mitteilen, ob nach den Angaben im Fragebogen die Voraussetzungen für die Teilnahme an Studienteil II erfüllt sind (dies wäre zum Beispiel nicht der Fall, wenn bei Ihnen erst vor kurzem eine präventive Darmspiegelung durchgeführt worden sein sollte). Alle in Frage kommenden Teilnehmerinnen und Teilnehmer werden per Zufallsverfahren in einen von zwei Studienarmen eingeteilt und per Post und ggf. telefonisch eingeladen, bei Interesse über das DARIO Studienzentrum einen Termin im Interdisziplinären Endoskopiezentrum (IEZ) des Universitätsklinikums Heidelberg oder evtl. in einem anderen von uns definierten Krankenhaus oder medizinischen Zentrum oder in einer gastroenterologischen Praxis im Rhein-Neckar-Gebiet (Heidelberg, Mannheim, Rhein-Neckar-Kreis) zu vereinbaren:

A) Wenn Sie in Studienarm A eingeteilt werden, bieten wir Ihnen an, eine präventive Koloskopie (komplette Darmspiegelung) am IEZ oder evtl. in einem anderen von uns definierten Krankenhaus oder medizinischen Zentrum oder in einer gastroenterologischen Praxis im Rhein-Neckar-Gebiet (Heidelberg, Mannheim, Rhein-Neckar-Kreis) auf unsere Kosten durchführen zu lassen.

B) Wenn Sie in Studienarm B eingeteilt werden, bieten wir an, alternativ eine präventive Koloskopie (komplette Darmspiegelung) oder eine Sigmoidoskopie („kleine Darmspiegelung“) am IEZ oder evtl. in einem anderen von uns definierten Krankenhaus oder medizinischen Zentrum oder in einer gastroenterologischen Praxis im Rhein-Neckar-Gebiet (Heidelberg, Mannheim, Rhein-Neckar-Kreis) auf unsere Kosten durchführen zu lassen.

Bei der Sigmoidoskopie kann nur ein Teil des Darms eingesehen werden, in dem etwa zwei Drittel aller Darmkrebserkrankungen auftreten. Dafür ist die Durchführung einfacher, es entfallen insbesondere die Abführmaßnahmen am Vortag. Durch die verschiedenen Angebote in den beiden Studienarmen möchten wir untersuchen, ob durch eine Erweiterung des Vorsorge-Angebots um die Sigmoidoskopie insgesamt noch bessere Vorsorge-Effekte erzielt werden können.

Wenn Sie an einer präventiven Darmspiegelung interessiert sind, vermitteln wir Ihnen einen Termin am IEZ, oder evtl. in einem anderen von uns definierten Krankenhaus oder medizinischen Zentrum oder in einer gastroenterologischen Praxis im Rhein-Neckar-Gebiet (Heidelberg, Mannheim, Rhein-Neckar-Kreis) bei dem Sie entsprechend Ihres Studienarms umfassende Informationen zur Koloskopie bzw. zur Koloskopie oder Sigmoidoskopie erhalten. Wenn Sie sich zu einer Darmspiegelung entschließen, bieten wir Ihnen eine kostenfreie Durchführung am IEZ oder evtl. in einem anderen von uns definierten Krankenhaus oder medizinischen Zentrum oder in einer gastroenterologischen Praxis im Rhein-Neckar-Gebiet (Heidelberg, Mannheim, Rhein-Neckar-Kreis) an. In diesem Fall bitten wir Sie auch um die Erlaubnis, die Befunde beim IEZ oder dem anderen Zentrum/der Praxis anzufordern. Über die Ergebnisse der Darmspiegelung werden Sie vom durchführenden Arzt informiert.

Auswertung und Datenschutz

Der ausgefüllte Fragebogen sowie sämtliche Befunde werden ausschließlich für die wissenschaftliche Erforschung von Fragestellungen zur Früherkennung und Vorsorge von Krebs und seinen Vorstufen verwendet. Alle Angaben aus Fragebogen und Befunden werden getrennt von Ihren Adressdaten (in pseudonymisierter Form) aufbewahrt. Zur Auswertung im Deutschen Krebsforschungszentrum oder durch Kooperationspartner werden nur pseudonymisierte Daten verwendet. "Pseudonymisiert" bedeutet, dass keine Angaben zu Namen, Initialen oder Adresse vorliegen, sondern nur ein Nummern- und/oder Buchstabencode verwendet wird. Eine Zuordnung der Daten zu einer bestimmten Person ist nur mit Hilfe eines Schlüssels möglich, der getrennt von Ihren personenbezogenen Daten verwaltet wird. **Wer den Fragebogen auswertet, weiß also nicht, von wem die Antworten gegeben wurden**. „Pseudonymisierung“ ist die Verarbeitung personenbezogener Daten in einer Weise, dass die personenbezogenen Daten ohne Hinzuziehung zusätzlicher Informationen („Schlüssel“) nicht mehr einer spezifischen betroffenen Person zugeordnet werden können. Diese zusätzlichen Informationen werden dabei gesondert aufbewahrt und unterliegen technischen und organisatorischen Maßnahmen, die gewährleisten, dass die personenbezogenen Daten nicht einer identifizierten oder identifizierbaren natürlichen Person zugewiesen werden. Pseudonymisierte Proben und Daten können auch zu Kooperationspartnern (z.B. Universitäten und Kliniken) national und international weitergeleitet, dort ausgewertet und langfristig gelagert werden. In diesem Zusammenhang möchten wir Sie auf ein möglicherweise niedrigeres Datenschutzniveau in Ländern außerhalb der Europäischen Union hinweisen, jedoch werden nur pseudonymisierte

Daten ohne eine Identifizierungsmöglichkeit weiter gegeben werden. Wenn alle für das Projekt erforderlichen Daten vollständig sind, spätestens jedoch nach 15 Monaten werden alle personenbezogenen Daten gelöscht. Eine nachträgliche Zuordnung der Daten zu einer bestimmten Person ist dann nicht mehr möglich (=Anonymisierung, d.h., das Verändern personenbezogener Daten in der Weise, dass die betroffene Person nicht mehr oder nur mit einem unverhältnismäßig großen Kosten- oder Zeitaufwand identifiziert werden kann). Nach 30 Jahren erfolgt eine Prüfung, ob die nur noch anonymisiert vorliegenden Daten weiter benötigt werden oder zu vernichten sind. Die Speicherung und Verarbeitung Ihrer Daten erfolgt unter strikter Beachtung der Landes- und Bundesdatenschutz-Gesetze und der Datenschutz-Grundverordnung der Europäischen Union (EU-DSGVO). Die „datenschutzrechtlichen Bestimmungen“ werden eingehalten. Zugang zu personenbezogenen Daten haben ausschließlich namentlich benannte, direkt mit der Durchführung der Studie betraute Mitarbeiter und Mitarbeiterinnen.

Freiwilligkeit der Teilnahme und Rücktrittsrecht

Ihre Teilnahme an dieser Studie ist freiwillig. Sie werden in diese Studie also nur dann einbezogen, wenn Sie dazu schriftlich Ihre Einwilligung geben. Sie können Ihr Einverständnis jederzeit schriftlich oder mündlich ohne Angabe von Gründen zurückziehen, ohne dass Ihnen hierdurch Nachteile entstehen. Die Teilnahme an der Studie beinhaltet keinerlei Verpflichtung zur Durchführung einer endoskopischen Untersuchung (Koloskopie oder Sigmoidoskopie). Im Falle des Widerrufs vor Anonymisierung haben Sie das Recht das Löschen Ihrer Daten zu fordern. In Ausnahmefällen kann es bei Durchführung einer endoskopischen Untersuchung zu sogenannten Zufallsbefunden kommen (z.B. nicht Polypen-bezogene Auffälligkeiten oder Verdacht auf andere Erkrankungen). Die Mitteilung von Zufallsbefunden können Sie ablehnen.

Welche weiteren Rechte haben Sie?

Auskunftsrecht: Sie können jederzeit Auskunft darüber verlangen welches Biomaterial und welche Daten bei uns über Sie verarbeitet werden.
Recht auf Berichtigung: Sie haben ein Recht auf Berichtigung und/oder Vervollständigung, sofern die verarbeiteten personenbezogenen Daten, die Sie betreffen, unrichtig oder unvollständig sind.
Recht auf Einschränkung der Verarbeitung: Sie können jederzeit die Einschränkung der Verarbeitung der Sie betreffenden personenbezogenen Daten verlangen.
Recht auf Löschung: Sie können jederzeit verlangen, dass die Sie betreffenden personenbezogenen Daten unverzüglich gelöscht werden.
Recht auf Datenübertragbarkeit: Sie haben das Recht, die Sie betreffenden personenbezogenen Daten in einem strukturierten, gängigen und maschinenlesbaren Format zu erhalten.
Recht auf Beschwerde bei einer Aufsichtsbehörde: Unbeschadet eines anderweitigen verwaltungsrechtlichen oder gerichtlichen Rechtsbehelfs steht Ihnen das Recht auf Beschwerde bei einer Aufsichtsbehörde zu, wenn Sie der Ansicht sind, dass die Verarbeitung der Sie betreffenden personenbezogenen Daten gegen die DSGVO verstößt.

Bei sonstigen rechtlichen Fragen und oder Anliegen zur Einhaltung der datenschutzrechtlichen Anforderungen können Sie sich gerne an den zuständigen Datenschutzbeauftragten wenden. Da die Studie am Deutschen Krebsforschungszentrum in Heidelberg durchgeführt wird, ist der Ansprechpartner für diese Belange wie folgt:

Die Verantwortliche Stelle für die Datenverarbeitung ist erreichbar unter:
Abt. Klinische Epidemiologie und Alternsforschung, Deutsches Krebsforschungszentrum
Im Neuenheimer Feld 581, 69120 Heidelberg
und Abt. Präventive Onkologie, Nationales Centrum für Tumorerkrankungen (NCT)
Im Neuenheimer Feld 460, 69120 Heidelberg
Telefon: 06221 - 56-34322
E-Mail: dario@nct-heidelberg.de

Der Datenschutzbeauftragte ist erreichbar unter:
Deutsches Krebsforschungszentrum (DKFZ)
Datenschutzbeauftragter
Im Neuenheimer Feld 280, 69120 Heidelberg
Telefon Nr.: 06221 42 -0
Email: datenschutz@dkfz-heidelberg.de

- Wir weisen Sie auf Ihr Recht hin, sich bei datenschutzrechtlichen Verstößen bei der Datenschutz-Aufsichtsbehörde zu beschweren.
- Für mögliche Beschwerden wenden Sie sich bitte, entsprechend Ihres Wohnortes, an die Aufsichtsbehörde Ihres Bundeslandes (siehe folgende Kontaktadresse):

Baden-Württemberg:
Landesbeauftragter für den Datenschutz und die Informationsfreiheit Baden-Württemberg
Postfach 10 29 32, 70025 Stuttgart
Königstraße 10a, 70173 Stuttgart
Tel.: 0711/61 55 41 – 0
Fax: 0711/61 55 41 – 15
E-Mail: poststelle@lfdi.bwl.de
http://www.baden-wuerttemberg.datenschutz.de

Die Teilnahme möglichst vieler Personen wird es ermöglichen, die Chancen der Früherkennung von Krebserkrankungen, insbesondere von Darmkrebs in Zukunft weiter zu verbessern. Wir möchten Sie daher sehr herzlich bitten, Ihr Einverständnis zur Teilnahme an der Studie durch Ihre Unterschrift auf der Einverständniserklärung festzuhalten.

Vielen Dank für Ihren wichtigen Beitrag zur Krebsforschung!

Prof. Dr. med. Hermann Brenner
Abt. Klinische Epidemiologie und Alternsforschung (DKFZ)
und Abt. Präventive Onkologie (NCT)
