# Supplementary material for: Effects of Alternative Offers of Screening Sigmoidoscopy and Colonoscopy on Utilization and Yield of Endoscopic Screening for Colorectal Neoplasms: Protocol of the DARIO Randomized Trial
Source: JMIR Res Protoc. 2020 Aug 5;9(8):e17516. doi: 10.2196/17516 (PMC7439136; doi:10.2196/17516)
Supplement: Multimedia Appendix 4 [file resprot_v9i8e17516_app4.pdf]

Abteilung Präventive Onkologie
Nationales Centrum für
Tumorerkrankungen (NCT)
Im Neuenheimer Feld 460
69120 Heidelberg
Telefon: 06221 - 56-34322

NATIONALES CENTRUM FÜR TUMORERKRANKUNGEN HEIDELBERG

getragen von:
Deutsches Krebsforschungszentrum
Universitätsklinikum Heidelberg
Thoraxklinik-Heidelberg
Deutsche Krebshilfe

DARIO: **Dar**mkrebsprävention – **I**nn**o**vative Wege am NCT

Teilnehmerinformation, Studienteil III

Sehr geehrte Teilnehmerin, sehr geehrter Teilnehmer,

für Ihre bisherige Teilnahme an der DARIO-Studie möchten wir uns sehr herzlich bei Ihnen bedanken. Wir hoffen, dass Ihre Teilnahme bislang positiv und reibungslos verlief. Mit diesem Schreiben möchten wir Sie nun bitten, auch an Teil III, dem letzten Teil der DARIO-Studie teilzunehmen.
Mit der Teilnahme an Studienteil III können Sie mit der Spende einer Blut-, Speichel-, Urin- und einer Stuhlprobe zur Entwicklung neuer Methoden für die Darmkrebsfrüherkennung beitragen, mit denen Krebserkrankungen durch die Analyse dieser Proben einfacher erkannt werden können. So wäre es zukünftig möglich, Personen auf Basis der Testergebnisse gezielter eine Darmspiegelung zu empfehlen.

Ablauf von Studienteil III

Wir möchten Sie in Studienteil III um eine Blutprobe (maximal 36 ml), eine Speichelprobe, eine Urin- und eine Stuhlprobe bitten. Dies wird ca. 20-30 Minuten Zeit in Anspruch nehmen.
Für die vorgesehene Stuhlprobe geben wir Ihnen ein Sammelpaket (Kit) mit einem beiliegenden Test auf Blut im Stuhl und mit ausführlicher Anleitung mit nach Hause, damit Sie sich hierfür zu Hause Zeit nehmen können. Das Probenbehältnis und den Stuhltest schicken Sie uns nach erfolgter Durchführung einfach entsprechend der Anleitung zurück. Wir bitten Sie jedoch zu beachten, dass Sie Ihre Stuhlprobe auf jeden Fall vor dem Abführen zur Darmspiegelung gewinnen sollten.

Welche Risiken sind damit verbunden?

Bei der Blutentnahme kann es, wie bei jeder Blutentnahme, in Ausnahmefällen zu einem Bluterguss kommen oder auch zu einer kurzfristigen Kreislaufreaktion (vegetative Reaktion). Äußerst selten sind Infektionen oder Nervenschädigungen.

Auswertung und Datenschutz

Ihre Proben werden ausschließlich zur wissenschaftlichen Erforschung von Krebserkrankungen verwendet: Zur wissenschaftlichen Grundlagenforschung, zur Erforschung von Vorsorgeuntersuchungen, Risikofaktoren und prognostischen Markern (einschließlich genetischer Faktoren), sowie zur Untersuchung der Entstehung, Diagnose und Therapie von Krebs- und anderen damit verbundenen Erkrankungen (z.B. Diabetes). Es kann sein, dass Ihre Proben und Daten auch für medizinische Forschungsfragen verwendet werden, die wir heute noch nicht absehen können. Deshalb werden an Ihren Biomaterialien möglicherweise auch genetische Untersuchungen, also Untersuchungen der Erbsubstanz, durchgeführt, und zwar unter Umständen auch eine Untersuchung Ihres gesamten Genoms. Ihre Biomaterialien und Daten sollen langfristig aufbewahrt und für die medizinische Forschung bereitgestellt werden (siehe 30-Jahres-Frist unten).

Bei jeder Erhebung, Speicherung und Übermittlung von Daten aus Ihren Biomaterialien im Rahmen von Forschungsprojekten bestehen Vertraulichkeitsrisiken (z.B. die Möglichkeit, Sie zu identifizieren), insbesondere im Hinblick auf die Information zu Ihrer Erbsubstanz. Diese Risiken lassen sich nicht völlig ausschließen und steigen, je mehr Daten miteinander verknüpft werden können, insbesondere auch dann, wenn Sie selbst (z.B. zur Ahnenforschung) genetische Daten im Internet veröffentlichen. Die Daten und die Auswertung Ihrer Proben können zu einem besseren Verständnis der Entstehung und Entwicklung von Krebs und anderen Erkrankungen, der Ansprechbarkeit auf die Behandlung, der Vorhersagbarkeit des Behandlungserfolges beitragen und möglicherweise zur Entwicklung von neuen Therapien und Diagnostika führen. Ein kommerzieller Nutzen der Ergebnisse kann daher nicht ausgeschlossen werden. Die Teilnahme an der Studie hat für Sie jedoch keinen kommerziellen Nutzen. Ein Teil der Proben wird ohne zeitliche Begrenzung nur mit der Identifikationsnummer gekennzeichnet aufbewahrt, um auch in Zukunft nach neuesten Erkenntnissen und mit verbesserten labortechnischen Möglichkeiten gezielte Analysen durchführen zu können.

Alle Proben werden getrennt von Ihren Adressdaten im Deutschen Krebsforschungszentrum in Heidelberg in pseudonymisierter (verschlüsselter) Form aufbewahrt und ausgewertet, ohne dass Ihre Identität dort erkannt werden kann (pseudonymisiert bedeutet, dass keine Angaben von Namen oder Initialen verwendet werden, sondern nur ein Nummern- und Buchstabencode). Wer den Fragebogen oder die Proben auswertet, weiß also nicht, von wem die Antworten gegeben wurden oder die Proben stammen. „Pseudonymisierung“ ist die Verarbeitung personenbezogener Daten in einer Weise, dass die personenbezogenen Daten ohne Hinzuziehung zusätzlicher Informationen („Schlüssel“) nicht mehr einer spezifischen betroffenen Person zugeordnet werden können. Diese zusätzlichen Informationen werden dabei gesondert aufbewahrt und unterliegen technischen und organisatorischen Maßnahmen, die gewährleisten, dass die personenbezogenen Daten nicht einer identifizierten oder identifizierbaren natürlichen Person zugewiesen werden. Pseudonymisierte Proben und Daten können auch zu Kooperationspartnern (z.B. Universitäten und Kliniken) national und international weitergeleitet, dort ausgewertet und langfristig gelagert werden. In diesem Zusammenhang möchten wir Sie auf ein möglicherweise niedrigeres Datenschutzniveau in Ländern außerhalb der Europäischen Union hinweisen, jedoch werden nur pseudonymisierte Daten ohne eine Identifizierungsmöglichkeit weiter gegeben werden. Falls Daten und Proben vor der vollständigen Anonymisierung weitergegeben werden, geschieht dies in doppelt pseudonymisierter Form (d.h. die weitergegebene Identifikationsnummer entspricht nicht der im DKFZ gespeicherten und verwendeten Hauptidentifikationsnummer). Im Zuge der wissenschaftlichen Auswertung Ihrer genetischen Daten, dazu gehören Ihre gesamten genetischen Daten, können diese auch in umfassende internationale Datenbanken eingegeben werden (z.B. die dbGaP-Datenbank in den USA oder die europäische EGA-Datenbank). Auch in diesem Falle werden Ihre Daten nur in verschlüsselter Form weitergegeben. In der Einwilligungserklärung bitten wir Sie um Ihre Zustimmung für diese wissenschaftliche Verwendung Ihrer Daten.

Außerhalb der Untersuchungen werden wir die ausgewerteten genetischen Daten nur zur wissenschaftlichen Publikation verwenden. Einige der hochrangigen wissenschaftlichen Zeitschriften verlangen für die Publikation dieser ausgewerteten pseudonymisierten genetischen Daten einen kontrollierten Zugang zu den Gesamtgenomdaten, um die wissenschaftliche Qualität der Publikation und die Forschungsergebnisse überprüfen zu können. Der Zugang zu den Daten wird durch spezielle Komitees kontrolliert. In der Einwilligungserklärung bitten wir Sie um Ihre Zustimmung für die wissenschaftliche Publikation Ihrer ausgewerteten, verschlüsselten genetischen Daten zu Forschungszwecken und die kontrollierte Bereitstellung Ihrer Gesamtgenomdaten für die Qualitätskontrolle wissenschaftlicher Zeitschriften.

Ihre personenidentifizierenden Daten (Name, Adresse) werden für bis zu 15 Monate im Deutschen Krebsforschungszentrum getrennt von den wissenschaftlichen Daten in einer separaten und zugangsgesicherten Datenbank gespeichert. Spätestens 15 Monate nach Ihrem Studieneintritt werden Ihre personenidentifizierenden Daten (Name, Adresse) gelöscht. Ab diesem Zeitpunkt sind die von Ihnen gespeicherten Daten und Proben anonymisiert (anonymisiert bedeutet, dass nur ein Nummern- oder Buchstabencode verwendet wird, bzw. das Verändern personenbezogener Daten in der Weise, dass die betroffene Person nicht mehr oder nur mit einem unverhältnismäßig großen Kosten- oder Zeitaufwand identifiziert werden kann). Ihre Daten und Proben können deshalb ab diesem Zeitpunkt nicht mehr personenbezogen gelöscht werden. Nach 30 Jahren erfolgt eine Prüfung, ob die nur noch anonymisiert vorliegenden Daten und Proben weiter benötigt werden oder zu vernichten sind.
Die Speicherung und Verarbeitung Ihrer Daten erfolgt unter strikter Beachtung der Landes- und Bundesdatenschutz-Gesetze und der DatenschutzGrundverordnung der Europäischen Union (EU-DSGVO). Die „datenschutzrechtlichen Bestimmungen“ werden eingehalten. Zugang zu den personenbezogenen Daten haben ausschließlich namentlich benannte, direkt mit der Durchführung der Studie betraute MitarbeiterInnen.

Freiwilligkeit der Teilnahme und Rücktrittsrecht

Auch die Teilnahme an Studienteil III ist freiwillig. Sie werden also nur dann einbezogen, wenn Sie dazu schriftlich Ihre Einwilligung und eine Übereignungsvereinbarung für die gespendeten Proben geben. Sie können Ihr Einverständnis schriftlich oder mündlich ohne Angabe von Gründen zurückziehen, ohne dass Ihnen hierdurch Nachteile entstehen. Die Teilnahme an Studienteil III ist nicht verpflichtend an die Durchführung einer endoskopischen Untersuchung (Koloskopie oder Sigmoidoskopie) gebunden. Im Falle des Widerrufs haben Sie bis zur Anonymisierung der Daten das Recht, das Löschen Ihrer Daten und die Vernichtung der Proben zu fordern.

Welche weiteren Rechte haben Sie?

Auskunftsrecht: Sie können jederzeit Auskunft darüber verlangen welches Biomaterial und welche Daten bei uns über Sie verarbeitet werden.
Recht auf Berichtigung: Sie haben ein Recht auf Berichtigung und/oder Vervollständigung, sofern die verarbeiteten personenbezogenen Daten, die Sie betreffen, unrichtig oder unvollständig sind.
Recht auf Einschränkung der Verarbeitung: Sie können jederzeit die Einschränkung der Verarbeitung der Sie betreffenden personenbezogenen Daten verlangen.
Recht auf Löschung: Sie können jederzeit verlangen, dass die Sie betreffenden personenbezogenen Daten unverzüglich gelöscht werden.
Recht auf Datenübertragbarkeit: Sie haben das Recht, die Sie betreffenden personenbezogenen Daten in einem strukturierten, gängigen und maschinenlesbaren Format zu erhalten.
Recht auf Beschwerde bei einer Aufsichtsbehörde: Unbeschadet eines anderweitigen verwaltungsrechtlichen oder gerichtlichen Rechtsbehelfs steht Ihnen das Recht auf Beschwerde bei einer Aufsichtsbehörde zu, wenn Sie der Ansicht sind, dass die Verarbeitung der Sie betreffenden personenbezogenen Daten gegen die DSGVO verstößt.

Bei sonstigen rechtlichen Fragen und oder Anliegen zur Einhaltung der datenschutzrechtlichen Anforderungen können Sie sich gerne an den zuständigen Datenschutzbeauftragten wenden. Da die Studie am Deutschen Krebsforschungszentrum in Heidelberg durchgeführt wird, ist der Ansprechpartner für diese Belange wie folgt:

Die Verantwortliche Stelle für die Datenverarbeitung ist erreichbar unter:
Abt. Klinische Epidemiologie und Alternsforschung, Deutsches Krebsforschungszentrum
Im Neuenheimer Feld 581, 69120 Heidelberg
und Abt. Präventive Onkologie, Nationales Centrum für Tumorerkrankungen (NCT)
Im Neuenheimer Feld 460, 69120 Heidelberg
Telefon: 06221 - 56-34322
E-Mail: dario@nct-heidelberg.de

Der Datenschutzbeauftragte ist erreichbar unter:
Deutsches Krebsforschungszentrum (DKFZ)
Datenschutzbeauftragter
Im Neuenheimer Feld 280, 69120 Heidelberg
Telefon Nr.: 06221 42 -0
Email: datenschutz@dkfz-heidelberg.de

- Wir weisen Sie auf Ihr Recht hin, sich bei datenschutzrechtlichen Verstößen bei der Datenschutz-Aufsichtsbehörde zu beschweren.
- Für mögliche Beschwerden wenden Sie sich bitte, entsprechend Ihres Wohnortes, an die Aufsichtsbehörde Ihres Bundeslandes (siehe folgende Kontaktadresse):

Baden-Württemberg:
Landesbeauftragter für den Datenschutz und die Informationsfreiheit Baden-Württemberg
Postfach 10 29 32, 70025 Stuttgart
Königstraße 10a, 70173 Stuttgart
Tel.: 0711/61 55 41 – 0
Fax: 0711/61 55 41 – 15
E-Mail: poststelle@lfdi.bwl.de
http://www.baden-wuerttemberg.datenschutz.de

Die Teilnahme möglichst vieler Personen wird es ermöglichen, die Chancen der Früherkennung von Krebserkrankungen, insbesondere von Darmkrebs in Zukunft weiter zu verbessern. Wir möchten Sie daher sehr herzlich bitten, Ihr Einverständnis zur Teilnahme an Studienteil III durch Ihre Unterschrift auf der Einverständniserklärung und der Übereignungsvereinbarung festzuhalten.

Vielen Dank für Ihren wichtigen Beitrag zur Krebsforschung!

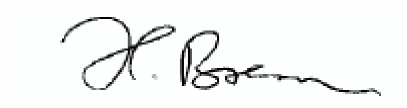

Prof. Dr. Hermann Brenner
Abt. Klinische Epidemiologie und Alternsforschung (DKFZ)
und Abteilung Präventive Onkologie (NCT)
