# Supplementary material for: Effects of Alternative Offers of Screening Sigmoidoscopy and Colonoscopy on Utilization and Yield of Endoscopic Screening for Colorectal Neoplasms: Protocol of the DARIO Randomized Trial
Source: JMIR Res Protoc. 2020 Aug 5;9(8):e17516. doi: 10.2196/17516 (PMC7439136; doi:10.2196/17516)
Supplement: Multimedia Appendix 5 [file resprot_v9i8e17516_app5.pdf]

Abteilung Präventive Onkologie
Nationales Centrum für
Tumorerkrankungen (NCT)
Im Neuenheimer Feld 460
69120 Heidelberg
Telefon: 06221 - 56-34322

NATIONALES CENTRUM FÜR TUMORERKRANKUNGEN HEIDELBERG

getragen von:
Deutsches Krebsforschungszentrum
Universitätsklinikum Heidelberg
Thoraxklinik-Heidelberg
Deutsche Krebshilfe

DARIO: **Dar**mkrebsprävention – **I**nn**o**vative Wege am NCT

Einwilligungserklärung Studienteile I und II

Ich bin verständlich über den Inhalt, die Vorgehensweise und den Zweck des oben genannten Forschungsvorhabens schriftlich und mündlich aufgeklärt worden. Ich habe den Text der Teilnehmerinformation, sowie die hier nachfolgend abgedruckte Datenschutzerklärung gelesen und verstanden. Ich hatte Gelegenheit, Fragen zu stellen und habe hierauf Antworten erhalten. Ich hatte ausreichend Zeit, mich für oder gegen eine Teilnahme zu entscheiden.

Mir ist bekannt, dass die Teilnahme freiwillig ist und dass ich die Einwilligung jederzeit ohne Angabe von Gründen widerrufen kann. Im Falle des Widerrufs habe ich das Recht, zusätzlich die Vernichtung des gesamten (Daten-) Materials schriftlich oder mündlich zu verlangen.

Ich willige ein, den Fragebogen mit Fragen zu früheren Vorsorgeuntersuchungen und meinen Lebensgewohnheiten auszufüllen (Studienteil I).

Ich willige ebenfalls ein, dass ich, bei Vorliegen der Voraussetzungen, nach Einsendung des Fragebogens zufällig in einen der zwei Studienarme eingeteilt werde, in denen mir eine kostenfreie endoskopische Vorsorgeuntersuchung des Darms im Interdisziplinären Endoskopiezentrum des Universitätsklinikums Heidelberg oder in einem anderen Krankenhaus oder medizinischen Zentrum oder einer gastroenterologischen Praxis im Rhein-Neckar-Gebiet (Heidelberg, Mannheim, Rhein-Neckar-Kreis) angeboten wird (Studienteil II). Falls ich dieses Angebot annehme, willige ich ein, dass die Ergebnisse dieser endoskopischen Untersuchung an die Mitarbeiter des Forschungsprojekts weiter gegeben werden. Falls ich mich nicht zu einer Annahme des Angebots entschließe, werden mir zu keinem Zeitpunkt der DARIO-Studie Nachteile entstehen. Ich bleibe dann immer noch Teilnehmer der DARIO-Studie.

Ich willige ein, dass meine personenidentifizierenden Daten (Name, Adresse) bis zum Abschluss der Datenerfassung im Deutschen Krebsforschungszentrum getrennt von den wissenschaftlichen Daten in einer separaten und zugangsgesicherten Datenbank gespeichert werden. Meine personenidentifizierenden Daten werden gelöscht, sobald die Datenerfassung abgeschlossen ist, spätestens jedoch 15 Monate nach Studieneintritt.

Bei Durchführung einer endoskopischen Untersuchung kann es in Ausnahmefällen zu sogenannten Zufallsbefunden kommen (z.B. nicht Polypen-bezogene Auffälligkeiten oder Verdacht auf andere Erkrankungen). Falls ich hierüber nicht informiert werden möchte, weise ich den Arzt im Vorgespräch zur endoskopischen Untersuchung darauf hin.

Datenschutz:
**Mir ist bekannt, dass bei dieser Studie personenbezogene Daten verarbeitet werden sollen. Die Verarbeitung der Daten erfolgt nach gesetzlichen Bestimmungen und setzt gemäß Art. 6 Abs. 1 lit. a der Datenschutz-Grundverordnung folgende Einwilligungserklärung voraus: Ich wurde darüber aufgeklärt und stimme freiwillig zu, dass meine im Rahmen der Studie ermittelten Daten/Krankheitsdaten, zu den in der Informationsschrift beschriebenen Zwecken ausschließlich in pseudonymisierter Form (Pseudonymisierung bedeutet Verschlüsselung von Daten ohne Namensnennung nur mit Nummern) aufgezeichnet und ausgewertet, sowie anonymisiert in wissenschaftlichen Fachzeitschriften veröffentlicht werden. Soweit erforderlich, dürfen die erhobenen Daten pseudonymisiert für zukünftige Forschungsvorhaben weitergegeben werden. Das heißt, die pseudonymisierten Daten können auch mit Kooperationspartnern national und international (z.B. Universitäten, Kliniken) ausgetauscht, dort ausgewertet und langfristig dort gelagert werden (siehe 30-Jahres-Frist unten). Auf ein möglicherweise niedrigeres Datenschutzniveau in Ländern außerhalb der Europäischen Union wurde ich hingewiesen. Dritte erhalten jedoch keinen Einblick in personenbezogene Unterlagen. Bei Inanspruchnahme der endoskopischen Vorsorge-Untersuchung erlaube ich den Mitarbeiterinnen und Mitarbeitern des Projektes, für die Studie notwendige Informationen zur durchgeführten endoskopischen Vorsorgeuntersuchung aus meinen ärztlichen Unterlagen zu entnehmen. Für diesen Vorgang entbinde ich die mich behandelnden Ärzte von ihrer ärztlichen Schweigepflicht. Meine personenidentifizierenden Daten werden gelöscht, sobald die Datenerfassung abgeschlossen ist, spätestens jedoch 15 Monate nach Studieneintritt. Nach 30 Jahren erfolgt eine Prüfung, ob meine nur noch anonymisiert vorliegenden Daten weiter benötigt werden oder zu vernichten sind.**

Aufgrund dieser Information erkläre ich mich freiwillig bereit an der oben genannten Studie teilzunehmen. Ein Exemplar der Teilnehmerinformation habe ich erhalten.

....

Bitte tragen Sie hier Ihren Namen in Druckbuchstaben ein

.... ➔ ....

Ort, Datum **Bitte unterschreiben Sie hier!**
