# Supplementary material for: Effects of Alternative Offers of Screening Sigmoidoscopy and Colonoscopy on Utilization and Yield of Endoscopic Screening for Colorectal Neoplasms: Protocol of the DARIO Randomized Trial
Source: JMIR Res Protoc. 2020 Aug 5;9(8):e17516. doi: 10.2196/17516 (PMC7439136; doi:10.2196/17516)
Supplement: Multimedia Appendix 6 [file resprot_v9i8e17516_app6.pdf]

Abteilung Präventive Onkologie
Nationales Centrum für
Tumorerkrankungen (NCT)
Im Neuenheimer Feld 460
69120 Heidelberg
Telefon: 06221 - 56-34322

NATIONALES CENTRUM FÜR TUMORERKRANKUNGEN HEIDELBERG

getragen von:
Deutsches Krebsforschungszentrum
Universitätsklinikum Heidelberg
Thoraxklinik-Heidelberg
Deutsche Krebshilfe

DARIO: **Dar**mkrebsprävention – **I**nn**o**vative Wege am NCT

Einwilligungserklärung, Studienteil III

Ich bin verständlich über den Inhalt, die Vorgehensweise und den Zweck des oben genannten Forschungsvorhabens schriftlich und mündlich aufgeklärt worden. Ich habe den Text der Teilnehmerinformation, sowie die hier nachfolgend abgedruckte Datenschutzerklärung gelesen und verstanden. Ich hatte Gelegenheit, Fragen zu stellen und habe hierauf Antworten erhalten. Ich hatte ausreichend Zeit, mich für oder gegen eine Teilnahme zu entscheiden.

Mir ist bekannt, dass die Teilnahme freiwillig ist und dass ich die Einwilligung jederzeit ohne Angabe von Gründen widerrufen kann. Im Falle des Widerrufs habe ich das Recht, zusätzlich die Vernichtung des gesamten (Daten-) Materials schriftlich oder mündlich zu verlangen.

Ich willige ein, je eine Blut- (maximal 36 ml), Stuhl-, Urin- und Speichelprobe sowie ggf. eine Probe eines bei der Endoskopie entnommenen Polypen für das Projekt zur Verfügung zu stellen. Die Proben werden, zusammen mit meinen Fragebogendaten und medizinischen Daten aus Studienteil I und II, ausschließlich zur Erforschung von Krebs und anderen damit verbundenen Erkrankungen verwendet.

Datenschutz:
**Mir ist bekannt, dass bei dieser Studie personenbezogene Daten verarbeitet werden sollen. Die Verarbeitung der Daten erfolgt nach gesetzlichen Bestimmungen und setzt gemäß Art. 6 Abs. 1 lit. a der Datenschutz-Grundverordnung folgende Einwilligungserklärung voraus: Ich wurde darüber aufgeklärt und stimme zu, dass meine im Rahmen der Studie ermittelten Daten/Krankheitsdaten, zu den in der Informationsschrift beschriebenen Zwecken ausschließlich in pseudonymisierter Form (Pseudonymisierung bedeutet Verschlüsselung von Daten ohne Namensnennung nur mit Nummern) aufgezeichnet und ausgewertet, sowie anonymisiert in wissenschaftlichen Fachzeitschriften veröffentlicht werden. Soweit erforderlich, dürfen die erhobenen Daten pseudonymisiert für zukünftige Forschungvorhaben weitergegeben werden. Das heißt, pseudonymisierte Proben und Daten können auch mit Kooperationspartnern national und international ausgetauscht, dort ausgewertet und langfristig dort gelagert werden (siehe 30-Jahres-Frist unten). Auf ein möglicherweise niedrigeres Datenschutzniveau in Ländern außerhalb der Europäischen Union wurde ich hingewiesen. Dritte erhalten jedoch keinen Einblick in personenbezogene Unterlagen. Meine personenidentifizierenden Daten werden gelöscht, sobald die Datenerfassung abgeschlossen ist, spätestens jedoch 15 Monate nach Studieneintritt. Nach 30 Jahren erfolgt eine Prüfung, ob meine nur noch anonymisiert vorliegenden Daten und Proben weiter benötigt werden oder zu vernichten sind.**

Aufgrund dieser Information erkläre ich mich freiwillig bereit an der oben genannten Studie teilzunehmen. Ein Exemplar der Teilnehmerinformation habe ich erhalten.

....
Bitte tragen Sie hier Ihren Namen in Druckbuchstaben ein

.... ➔...
Ort, Datum **Bitte unterschreiben Sie hier!**

Übereignungsvereinbarung

Zwischen dem **Deutschen Krebsforschungszentrum**, handelnd für die Abteilung Präventive Onkologie, vertreten durch Herrn Prof. Dr. H. Brenner, und mir als Studienteilnehmer wird folgendes vereinbart:

1. Ich übertrage dem Deutschen Krebsforschungszentrum unentgeltlich das Eigentum an folgenden Körpermaterialien:
   - Speichelproben
   - ca. 36 ml Blut
   - Urinproben
   - Stuhlproben
   - Möglicherweise Gewebeproben aus dem Dickdarm (die routinemäßig bei der Abtragung von Darmpolypen bei der endoskopischen Untersuchung anfallen könnten).

   Ich habe das Recht, bis zum Zeitpunkt der Anonymisierung jederzeit schriftlich oder telefonisch die Vernichtung der übertragenen Materialien und der daraus bestimmten Laborergebnisse zu fordern (s. Kontaktdaten im Briefkopf der Teilnehmerinformation).

2. Das Deutsche Krebsforschungszentrum und beteiligte Forschungspartner können die Körpermaterialien bzw. Daten langfristig in anonymisierter Form aufbewahren (anonymisiert bedeutet, dass nur ein Nummern- oder Buchstabencode verwendet wird. Eine nachträgliche Zuordnung der Proben/ Daten zu einer bestimmten Person ist nicht mehr möglich). Nach 30 Jahren erfolgt eine Prüfung, ob meine nur noch anonymisiert vorliegenden Daten und Proben weiter benötigt werden oder zu vernichten sind. Das Deutsche Krebsforschungszentrum und beteiligte Forschungspartner werden die Körpermaterialien ausschließlich zu folgenden Zwecken verwenden: zur wissenschaftlichen Grundlagenforschung, zur Erforschung von Vorsorgeuntersuchungen, Risikofaktoren und prognostischen Markern (einschließlich genetischer Faktoren), sowie zur Untersuchung der Entstehung, Diagnose und Therapie von Krebs- und anderen damit verbundenen Erkrankungen. Ich willige ein, dass molekulargenetische Untersuchungen durchgeführt werden. Hierbei können Gene oder DNA-Abschnitte durch verschiedene Methoden auf das Vorhandensein von Mutationen untersucht, sowie auch das ganze Genom sequenziert werden. Die daraus gewonnenen Erkenntnisse können einen großen Einfluss auf Therapie und Prognose bei Krebs- und anderen damit verbundenen Erkrankungen haben. Meine Daten und die Auswertung meiner Proben können zu einem besseren Verständnis der Entstehung und Entwicklung von Krebs und anderen Erkrankungen, der Ansprechbarkeit auf die Behandlung und der Vorhersagbarkeit des Behandlungserfolges beitragen und möglicherweise zur Entwicklung von neuen Therapien und Diagnostika führen. Ein kommerzieller Nutzen der Ergebnisse kann daher nicht ausgeschlossen werden.

3. Ich bin darüber informiert, dass die Teilnahme an der Studie für mich keinen persönlichen Vorteil oder kommerziellen Nutzen beinhaltet. Alle Rechte, die mit der Entwicklung neuer Therapien und Diagnostika, neuen Erkenntnissen oder der Entstehung schützenswerten Eigentums verbunden sind, übertrage ich dem DKFZ und den beteiligten Forschungspartnern. Ich willige weiterhin ein, dass meine gesamten genetischen Daten im Zuge der wissenschaftlichen Auswertung in umfassenden internationalen Datenbanken und in wissenschaftlichen Fachzeitschriften in anonymisierter Form und mit kontrolliertem Zugang zu den Gesamtgenomdaten veröffentlicht werden (wie in der Teilnehmerinformation beschrieben). Die Proben und Daten können zu internationalen und nationalen Institutionen und Kooperationspartnern (z.B. Universitäten, Kliniken) zur Analyse verschickt bzw. dort auch langfristig gelagert werden. Nach 30 Jahren erfolgt eine Prüfung, ob meine nur noch anonymisiert vorliegenden Daten und Proben weiter benötigt werden oder zu vernichten sind. Die Anonymität des Spenders bleibt gegenüber Dritten gewahrt.

➔ ....

Bitte unterschreiben Sie hier!

Noch einmal ganz herzlichen Dank für Ihre Teilnahme!

....

Prof. Dr. H. Brenner
Abteilung Klinische Epidemiologie
und Alternsforschung (DKFZ) und
Abteilung Präventive Onkologie (NCT)
Im Neuenheimer Feld 460
69120 Heidelberg
