# Supplementary material for: Effects of Alternative Offers of Screening Sigmoidoscopy and Colonoscopy on Utilization and Yield of Endoscopic Screening for Colorectal Neoplasms: Protocol of the DARIO Randomized Trial
Source: JMIR Res Protoc. 2020 Aug 5;9(8):e17516. doi: 10.2196/17516 (PMC7439136; doi:10.2196/17516)
Supplement: Multimedia Appendix 7 [file resprot_v9i8e17516_app7.pdf]

Darmkrebsprävention – **I**nn**o**vative Wege am NCT

Fragebogen für Teilnehmer

Bitte tragen Sie hier für eventuelle Rückfragen zum Fragebogen Ihre E-Mail-Adresse und/oder Ihre Telefonnummer ein.

☐ **Frau** ☐ **Herr**

Vorname: |__|__|__|__|__|__|__|__|__|__|__|__|__|__|__|__|

Nachname |__|__|__|__|__|__|__|__|__|__|__|__|__|__|__|__|

Geburtsdatum: |__|__|.|__|__|.|__|__|__|__|

Straße: |__|__|__|__| |__|__|__|__| |__|__|__|__|

PLZ, Wohnort: |__|__|__|__|__| |__|__|__|__|__|__|__|__|__|__|__|

Telefonnummer: .... / ....

E-Mail-Adresse ....@.........................

(für eventuelle Rückfragen zum Fragebogen)

Lesen Sie sich diese kurze Anleitung bitte vor dem Ausfüllen des Fragebogens durch!

Ein Beispiel zum Ausfüllen des Fragebogens:

1. Hatte Ihre Mutter / Ihr Vater jemals Darmkrebs?

Mutter:	☒ NEIN	☐ JA	☐ weiß nicht
Vater:	☐ NEIN	☒ JA	☐ weiß nicht

Falls JA, *in welchem Alter wurde die Erkrankung diagnostiziert?*

Mutter:	☐ <50	☐ 50-59	☐ 60-69	☐ 70+
Vater:	☒ <50	☐ 50-59	☐ 60-69	☐ 70+

Der Fragebogen wird maschinell verarbeitet (eingescannt). Machen Sie bitte daher nach Möglichkeit keine Angaben außerhalb dafür vorgesehenen Kästchen und Felder. Korrigieren Sie falsche Angaben, wie z.B. falsch angekreuzte Felder, deutlich durch eindeutiges Durchstreichen oder indem Sie das Feld vollkommen schwärzen.

Bitte verwenden Sie zum Ausfüllen idealerweise einen **schwarzen** Kugelschreiber oder Fineliner. **Bitte keinen dünnen blauen Kugelschreiber und auf keinen Fall einen grünen Stift verwenden!**

T

Heutiges Datum: |__|__| . |__|__| . |__|__|__|__|
Tag Monat Jahr

A. Fragen zu Ihrer Gesundheit und Vorsorgeuntersuchung

T

1. Wurde bei Ihnen jemals eine der folgenden Erkrankungen von einem Arzt festgestellt?

*Bitte kreuzen Sie bei jeder Erkankung **NEIN** oder **JA** an!*
***Falls JA**: In welchem Jahr erstmals?*

Herzinfarkt	☐ NEIN	☐ JA	|__|__|__|__| Jahr
Schlaganfall	☐ NEIN	☐ JA	|__|__|__|__| Jahr
Bluthochdruck	☐ NEIN	☐ JA	|__|__|__|__| Jahr
Diabetes (Zuckerkrankheit)	☐ NEIN	☐ JA	|__|__|__|__| Jahr
Chronisch entzündliche Darmerkrankung (z.B. Morbus Crohn, Colitis Ulcerosa)	☐ NEIN	☐ JA	|__|__|__|__| Jahr
Familiäre adenomatöse Polyposis (FAP)	☐ NEIN	☐ JA	|__|__|__|__| Jahr
Lynch Syndrom	☐ NEIN	☐ JA	|__|__|__|__| Jahr
Darmkrebs	☐ NEIN	☐ JA	|__|__|__|__| Jahr
Krebs (außer Darmkrebs)	☐ NEIN	☐ JA	|__|__|__|__| Jahr

Falls JA: Welche Krebserkrankung(en) war(en) das?

1. ....

2. ....

3. ....

T

T T

2. Wurde bei Ihnen jemals eine der folgenden Untersuchungen durchgeführt?

			Falls JA, wann zuerst (Jahr)	Falls JA, wann zuletzt (Jahr)	Wie oft insgesamt
Test auf Blut im Stuhl	☐ NEIN	☐ JA	|__|__|__|__|	|__|__|__|__|	|__|__| mal
Röntgenuntersuchung des Darms (Kontrast-mitteleinlauf, KME)	☐ NEIN	☐ JA	|__|__|__|__|	|__|__|__|__|	|__|__| mal
Darmspiegelung	☐ NEIN	☐ JA	|__|__|__|__|	|__|__|__|__|	|__|__| mal

➪ ***Falls NEIN**, bitte weiter mit Frage 3*

Falls jemals mindestens *eine* Darmspiegelung durchgeführt wurde:

2.1 Warum wurde die *erste* Darmspiegelung durchgeführt? *Bitte Hauptgrund ankreuzen!*

☐ Beschwerden wie Bauchschmerzen, Bauchkrämpfe, veränderte Stuhlgewohnheiten

☐ positiver Test auf Blut im Stuhl

☐ familiäre Vorgeschichte

☐ Vorsorge, eigener Wunsch

Wurden bei dieser **ersten** Darmspiegelung **Polypen** aus dem Darm entfernt? ☐ NEIN ☐ JA ☐ weiß nicht

Falls *mehr* als eine Darmspiegelung durchgeführt wurde:

2.2 Warum wurde die letzte Darmspiegelung durchgeführt? *Bitte Hauptgrund ankreuzen!*

☐ Beschwerden wie Bauchschmerzen, Bauchkrämpfe, veränderte Stuhlgewohnheiten

☐ positiver Test auf Blut im Stuhl

☐ familiäre Vorgeschichte

☐ Vorsorge, eigener Wunsch

Wurden bei der **letzten** Darmspiegelung **Polypen** aus dem Darm entfernt? ☐ NEIN ☐ JA ☐ weiß nicht

T T

T

3. Wurde bei Ihnen jemals eine der folgenden sonstigen Vorsorgeuntersuchungen durchgeführt?

*Bitte kreuzen Sie bei jeder Vorsorgeuntersuchung **NEIN** oder **JA** an!*

Falls JA: Wann **zuletzt**?

T

Allgemeiner Gesundheits-Check-Up (ab 35 Jahren angeboten)	☐ NEIN	☐ JA	|__|__|__|__| (Jahr)
Hautkrebs-Früherkennung	☐ NEIN	☐ JA	|__|__|__|__| (Jahr)
Mammographie	☐ NEIN	☐ JA	|__|__|__|__| (Jahr)
Vorsorgeuntersuchung auf Gebärmutterhalskrebs (z.B. Paptest, Abstrich)	☐ NEIN	☐ JA	|__|__|__|__| (Jahr)
Vorsorgeuntersuchung auf Prostatakrebs (PSA-Test)	☐ NEIN	☐ JA	|__|__|__|__| (Jahr)

T

4. Wie würden Sie Ihren Gesundheitszustand im Allgemeinen beschreiben?

Bitte kreuzen Sie nur ein Kästchen an!

☐ ausgezeichnet ☐ sehr gut ☐ gut ☐ weniger gut ☐ schlecht

5. Wie schätzen Sie Ihr Darmkrebsrisiko im Vergleich zu gleichaltrigen Personen ein?

Bitte kreuzen Sie nur ein Kästchen an!

☐ wesentlich geringer ☐ geringer ☐ gleich ☐ erhöht ☐ wesentlich erhöht

T

B. Fragen zu Erkrankungen bei Verwandten ersten Grades

Bitte beantworten Sie die folgende Frage gesondert für Ihre Mutter, Ihren Vater und Ihre Geschwister:

6. Hatte(n) Ihre Mutter, Ihr Vater oder mindestens eines Ihrer Geschwister jemals Darmkrebs?

Mutter: ☐ NEIN ☐ JA ☐ weiß nicht

Vater: ☐ NEIN ☐ JA ☐ weiß nicht

Geschwister: ☐ NEIN ☐ JA ☐ weiß nicht

☐ NEIN, keine Geschwister vorhanden

➪ ***Falls überall NEIN,*** *bitte weiter mit Frage 7*

➪ ***Falls JA,*** *in welchem Alter wurde der Darmkrebs diagnostiziert?*

	<50	*50-59*	*60-69*	*70-79*	*80 oder älter*	*weiß nicht*
Mutter:	☐	☐	☐	☐	☐	☐
Vater:	☐	☐	☐	☐	☐	☐
Geschwister:	☐	☐	☐	☐	☐	☐

(Wenn bei mehr als einem Geschwisterteil Darmkrebs diagnostiziert wurde, geben Sie bitte das Geschwisterteil mit dem niedrigsten Alter bei Diagnosestellung an.)

T

T

C. Fragen zu Erkrankungen bei Verwandten zweiten Grades

Bitte beantworten Sie die folgende Frage gesondert für Ihre Großeltern und die Geschwister Ihrer Eltern:

7. Hatte einer Ihrer Großeltern oder mindestens ein Geschwisterteil Ihrer Eltern jemals Darmkrebs?

Großmutter:	☐ NEIN	☐ JA	☐ weiß nicht
Großvater:	☐ NEIN	☐ JA	☐ weiß nicht
Geschwister Ihrer Eltern:	☐ NEIN	☐ JA	☐ weiß nicht
	☐ NEIN, keine Geschwister der Eltern vorhanden		

➪ ***Falls überall NEIN,*** *bitte weiter mit Frage 8*

➪ ***Falls JA,*** *in welchem Alter wurde der Darmkrebs diagnostiziert?*

(Wenn bei mehr als einer Großmutter, einem Großvater oder einem Geschwisterteil Ihrer Eltern Darmkrebs diagnostiziert wurde, geben Sie bitte jeweils den Verwandten mit dem niedrigsten Alter bei Diagnosestellung an.)

	<50	*50-59*	*60-69*	*70-79*	*80 oder älter*	*weiß nicht*
Großmutter:	☐	☐	☐	☐	☐	☐
Großvater:	☐	☐	☐	☐	☐	☐
Geschwister Ihrer Eltern:	☐	☐	☐	☐	☐	☐

T

D. Fragen zu Ihren Lebensgewohnheiten

8. Haben Sie in Ihrem Leben jemals regelmäßig, d.h. täglich über mindestens 1 Jahr, geraucht?

☐ NEIN ➪ *Bitte weiter mit Frage 11*

☐ JA

Falls JA: Rauchen Sie **derzeit**?

☐ NEIN

☐ JA, und zwar durchschnittlich pro Tag

|__|__| Zigaretten

|__|__| Zigarren

|__|__| Pfeifen

*Tragen Sie bitte bei den Tabakwaren, die Sie **nicht** rauchen, eine **0** ein.*

T

T

9. In welchem Alter haben Sie begonnen regelmäßig zu rauchen?

mit |__|__| Jahren

Falls Sie derzeit nicht mehr rauchen:
In welchem Alter haben Sie aufgehört? mit |__|__| Jahren

T

10. Wie viele Jahre haben Sie insgesamt regelmäßig geraucht?

Insgesamt etwa |__|__| Jahre

Wie viel haben Sie in dieser Zeit durchschnittlich pro Tag geraucht? |__|__| Zigaretten

*Tragen Sie bitte bei den Tabakwaren, die Sie **nicht** geraucht haben, eine **0** ein.* |__|__| Zigarren

|__|__| Pfeifen

11. An wie vielen Tagen pro Woche haben Sie in den letzten 12 Monaten üblicherweise Alkohol getrunken?

An |__| Tagen pro Woche

12. Wie viele alkoholische Getränke haben Sie in den letzten 12 Monaten durchschnittlich pro Woche getrunken?

*Tragen Sie bitte bei den alkoholischen Getränken, die Sie **nicht** getrunken haben, eine **0** ein!*

In den letzten 12 Monaten durchschnittlich pro Woche	**Bier** (Flasche zu 0,5l)	**Wein / Sekt** (Gläser zu ¼l)	**Schnaps** (Gläschen zu 2cl)
	|__|__| *Flaschen / Woche*	|__|__| *Gläser / Woche*	|__|__| *Gläschen / Woche*

T

B

T T

13. Wie oft haben Sie in den letzten 12 Monaten durchschnittlich folgende Nahrungsmittel und Getränke zu sich genommen?

	Nie	1-3x pro Monat	1x pro Woche	2-6x pro Woche	1x pro Tag	2-3x pro Tag	4 oder mehr pro Tag
Rotes Fleisch (Schwein, Rind usw.)	☐	☐	☐	☐	☐	☐	☐
Geflügel	☐	☐	☐	☐	☐	☐	☐
Fisch	☐	☐	☐	☐	☐	☐	☐
Schwein, Rind: Wurstwaren oder Salami	☐	☐	☐	☐	☐	☐	☐
Geflügel: Wurstwaren oder Salami	☐	☐	☐	☐	☐	☐	☐
Eier	☐	☐	☐	☐	☐	☐	☐
Vollkornprodukte (Brot, Müsli)	☐	☐	☐	☐	☐	☐	☐
Weißmehlprodukte (Brot, Toast, Brezeln, Nudeln, Reis)	☐	☐	☐	☐	☐	☐	☐
Gemüse oder Salat	☐	☐	☐	☐	☐	☐	☐
Obst oder Obstsäfte	☐	☐	☐	☐	☐	☐	☐
Olivenöl oder Rapsöl	☐	☐	☐	☐	☐	☐	☐
Butter	☐	☐	☐	☐	☐	☐	☐
Margarine	☐	☐	☐	☐	☐	☐	☐
Kartoffeln	☐	☐	☐	☐	☐	☐	☐
Frittiertes (Pommes, Chips)	☐	☐	☐	☐	☐	☐	☐
Hülsenfrüchte (Bohnen, Linsen, Erbsen usw.)	☐	☐	☐	☐	☐	☐	☐
Süßwaren	☐	☐	☐	☐	☐	☐	☐
Nüsse oder Saaten	☐	☐	☐	☐	☐	☐	☐
Kaffee	☐	☐	☐	☐	☐	☐	☐
Käse oder Quark	☐	☐	☐	☐	☐	☐	☐
Milch oder Joghurt	☐	☐	☐	☐	☐	☐	☐

T

T

14. Wie lange praktizieren Sie diese Art der Ernährung bereits?

|__|__| Jahre

15. Welche Art von Milch und Joghurt verzehren Sie überwiegend?

☐ Vollfett (3,5%) ☐ Halbfett (1,5%) ☐ ohne Fett (0,1%)

T

16. Wie viele Stunden pro Woche haben Sie sich in den letzten 12 Monaten durchschnittlich folgendermaßen körperlich betätigt?

Bitte trennen Sie hierbei – so gut es geht – zwischen leichten Betätigungen, leichterer Arbeit, körperlich anstrengender Arbeit und sportlichen Aktivitäten.
Falls Sie eine Tätigkeit nicht ausgeübt haben (z.B. Sport), tragen Sie bitte eine 0 ein.

**In den letzten 12 Monaten**	Stunden pro Woche (durchschnittlich)
**Körperlich anstrengende Arbeit**<br>(z.B. in der Landwirtschaft, als Bauarbeiter, in der Alten- und Krankenpflege ...)	|__|__|
**Körperlich anstrengende sportliche Aktivität**<br>(z.B. Fußball, Schwimmen, Skifahren, sportliches Radfahren, Bergsteigen, Joggen …)	|__|__|
**Leichtere, vorwiegend gehende oder stehende Arbeit**<br>(z.B. Hausarbeit, Gartenarbeit, Verkäufer …)	|__|__|
**Leichte Betätigung**<br>(z.B. zu Fuß zur Arbeit / zum Einkaufen gehen, Spaziergänge, Radfahren …)	|__|__|

T T

E. Fragen zur Medikamenteneinnahme

T

17. Haben Sie jemals gelegentlich oder regelmäßig Acetylsalicylsäure als Medikament zur Blutverdünnung (z.B. Aspirin 100, Aspirin 300, Aspirin protect 100, ASS 100, HerzASS, Godamed 100 TAH) eingenommen?

☐ NEIN

☐ JA, gelegentlich

☐ JA, regelmäßig (mehr als einmal pro Woche)

Falls regelmäßig: Wie viele Jahre insgesamt? |__|__| Jahre

Verwenden Sie **derzeit regelmäßig** Acetylsalicylsäure als Medikament zur Blutverdünnung?

☐ JA, welche(s) Mittel?

....

☐ NEIN, in welchem Jahr zuletzt? |__|__|__|__| *(Jahr)*

T

18. Haben Sie jemals gelegentlich oder regelmäßig Schmerzmittel oder Rheumamittel (z.B. Aspirin 500, ASS 500, Voltaren, Paracetamol, Dolormin, Ibuprofen o.Ä.) eingenommen?

☐ NEIN

☐ JA, gelegentlich

☐ JA, regelmäßig (mehr als einmal pro Woche)

Falls regelmäßig: Wie viele Jahre insgesamt? |__|__| Jahre

Verwenden Sie **derzeit regelmäßig** ein Schmerz- oder Rheumamittel?

☐ JA, welche(s) Mittel? T

1. ....

2. ....

☐ NEIN, in welchem Jahr zuletzt? |__|__|__|__| *(Jahr)*

T

T

F. Angaben zu Ihrer Person

19. Welche Nationalität haben Sie?

☐ deutsch ☐ sonstige: ....

20. In welchem Land sind Sie geboren?

☐ Deutschland ☐ anderes Land: ....

21. Welchen Familienstand haben Sie aktuell?

☐ ledig ☐ verheiratet ☐ geschieden ☐ verwitwet

22. Wie viele Personen wohnen insgesamt in Ihrem Haushalt (Sie selbst eingeschlossen)?

|__|__| Personen

23. Wie viel wiegen Sie derzeit und wie viel wogen Sie früher?

derzeit	etwa |__|__|__| kg
mit 40 Jahren	etwa |__|__|__| kg
mit 30 Jahren	etwa |__|__|__| kg
mit 20 Jahren	etwa |__|__|__| kg

T

24. Wie groß sind Sie?

|__|__|__| cm

T

T

25. Was ist Ihr höchster Schulabschluss (ohne Berufs- und Hochschulausbildung)?

☐ kein Abschluss

☐ Hauptschule / Volksschule

☐ Mittlere Reife

☐ Handelsschule

☐ Fachhochschulreife

☐ Abitur

☐ anderer ....

T

G. Die folgenden Fragen wenden sich nur an Frauen

26. Wie alt waren Sie bei der ersten Regelblutung?

|__|__| Jahre

T

27. Haben Sie Ihre Regelblutung noch?

☐ JA

☐ JA, aber die Regelblutungen sind unregelmäßig

☐ NEIN, die Regelblutungen haben aufgehört im Alter von |__|__| Jahren

28. Welche Ursache führte bei Ihnen zum Ausbleiben der Regel?

☐ natürliches Ende der Regel

☐ Entfernung beider Eierstöcke oder der Gebärmutter

☐ anderer Grund

T

T

29. Haben Sie jemals ein Kind geboren?

☐ NEIN ➪ *Bitte weiter mit Frage 29*

☐ JA, und zwar |__|__| *(Anzahl der Kinder)*

Falls JA, wie alt waren Sie bei der Geburt Ihres ersten Kindes? |__|__| Jahre

Haben Sie jemals gestillt?

☐ NEIN ➪ *Bitte weiter mit Frage 29*

☐ JA

Falls JA, wie viele Monate insgesamt (alle Kinder zusammen)? |__|__| Monate

T

30. Haben Sie jemals eine Hormon-Ersatz-Therapie (Tabletten, Pflaster, Gel, Spritzen, Creme, Tropfen, Spray etc.) durchgeführt, die Ihnen von einem Arzt verschrieben wurde?

(Zur Linderung von Wechseljahresbeschwerden oder zur Vorbeugung bestimmter Erkrankungen (z.B. Osteoporose) können Hormon-Ersatz-Präparate (z.B. Östrogene) angewendet werden. Manche Frauen bekommen diese Präparate auch nach der Entfernung beider Eierstöcke verschrieben.
*Diese Frage bezieht sich **nicht** auf pflanzliche Präparate oder homöopathische Mittel.)*

☐ NEIN

☐ JA

Falls JA: Wie viele Jahre insgesamt? |__|__| Jahre

Verwenden Sie **derzeit** ein solches Präparat?

☐ JA

☐ NEIN, in welchem Jahr zuletzt? |__|__|__|__| *(Jahreszahl)*

T T

Herzlichen Dank für Ihre Teilnahme!

**Bitte vergewissern Sie sich vor dem Abgeben,
dass Sie alle Fragen vollständig beantwortet haben.**
