# Supplementary material for: Effects of Alternative Offers of Screening Sigmoidoscopy and Colonoscopy on Utilization and Yield of Endoscopic Screening for Colorectal Neoplasms: Protocol of the DARIO Randomized Trial
Source: JMIR Res Protoc. 2020 Aug 5;9(8):e17516. doi: 10.2196/17516 (PMC7439136; doi:10.2196/17516)
Supplement: Multimedia Appendix 8 [file resprot_v9i8e17516_app8.pdf]

NATIONALES CENTRUM FÜR TUMORERKRANKUNGEN HEIDELBERG

getragen von:
Deutsches Krebsforschungszentrum
Universitätsklinikum Heidelberg
Thoraxklinik-Heidelberg
Deutsche Krebshilfe

NCT | Im Neuenheimer Feld 460 (G110) | D-69120 Heidelberg

Prof. Dr. med. Hermann Brenner
Leiter Abteilung Präventive Onkologie

Im Neuenheimer Feld 460
D-69120 Heidelberg

DARIO Studie
Telefon: 06221 56 34322
Telefax: 06221 56-5231
Email: dario@nct-heidelberg.de

Heidelberg,

DARIO Studie:
Darmkrebsprävention – Innovative Wege am NCT

Einladung zur Teilnahme an der DARIO Studie

Sehr geehrte Frau/Herr ….,

das Nationale Centrum für Tumorerkrankungen (NCT) und das Universitätsklinikum Heidelberg möchten Sie mit diesem Brief, gemeinsam mit ca. 12.000 weiteren, zufällig ausgewählten Personen im Alter von 50-54 Jahren, sehr herzlich zur Teilnahme an der DARIO Studie einladen. Falls Sie gerade in diesem Jahr Ihren 55. Geburtstag gefeiert haben, sind Sie trotzdem noch in unserer Studie herzlich willkommen. Ihre Kontaktdaten wurden uns vom Einwohnermeldeamt aufgrund einer nach §46 des Bundesmeldegesetzes (BMG) genehmigten Stichprobenziehung für wissenschaftliche Umfragen zur Verfügung gestellt.

Teil I der Studie besteht aus der Beantwortung des beigefügten kurzen Fragebogens und soll vor allem dazu beitragen, mehr über die Inanspruchnahme von Darmkrebsvorsorge-Untersuchungen zu erfahren. Durch das Ausfüllen und Zurücksenden des Fragebogens leisten Sie bereits einen wichtigen Beitrag.

In Teil II der Studie bieten wir Personen, die den Fragebogen ausgefüllt haben und bei denen bisher keine Darmspiegelung durchgeführt wurde, eine kostenfreie Darmspiegelung am Interdisziplinären Endoskopiezentrum (IEZ) des Universitätsklinikums Heidelberg an, einem ausgewiesenen Zentrum des Universitätsklinikums Heidelberg unter Leitung von Herrn Prof. Dr. med. Peter Sauer. Falls Sie dieses Angebot wahrnehmen möchten, werden Sie zunächst zu einem persönlichen Gespräch in das IEZ eingeladen, bei dem Sie nähere Informationen über die bestehenden Vorsorge-Möglichkeiten erhalten. Sie werden dabei zufällig einer von zwei Gruppen zugeteilt. Teilnehmer beider Gruppen erhalten das Angebot einer „großen Darmspiegelung“, der Koloskopie; Teilnehmer der zweiten Gruppe haben die Wahl zwischen dem Angebot der Koloskopie und der

Nationales Centrum für Tumorerkrankungen (NCT) Heidelberg
Im Neuenheimer Feld 460
D-69120 Heidelberg
www.nct-heidelberg.de

Onkologische Sprechstunden
Allgemeine Onkologie
CUP (Cancer of Unknown Primary)
Dermatologische Tumoren
Gastrointestinale Tumoren
Gynäkologische Tumoren
Gynäkologisch-genetische Sprechstd.
Kopf-Hals Tumoren
Kinderonkologie
Leukämie
Lymphome
Myelome
Neuroendokrine Tumoren
Neuroonkologie
Radioonkologie
Sarkome
Thorakale Tumoren
Urologische Tumoren

Beratungen
Krebsinformationsdienst (KID)
Ernährung
Psychoonkologie
Sozialdienst
Bewegung und Krebs

Geschäftsführende Direktoren
Prof. Dr. Stefan Fröhling (komm.)
Präzisionsonkologie, Deutsches Krebsforschungszentrum (DKFZ)

Prof. Dr. Dirk Jäger
Medizinische Onkologie, Universitätsklinikum Heidelberg (UKHD)

Stellvertretende Direktoren
Prof. Dr. Peter Lichter
Molekulare Genetik, Deutsches Krebsforschungszentrum (DKFZ)

Prof. Dr. Dr. Jürgen Debus
Radioonkologie, Universitätsklinikum Heidelberg (UKHD)

NATIONALES CENTRUM
FÜR TUMORERKRANKUNGEN
HEIDELBERG

getragen von:
Deutsches Krebsforschungszentrum
Universitätsklinikum Heidelberg
Thoraxklinik-Heidelberg
Deutsche Krebshilfe

Sigmoidoskopie, der sogenannten „kleinen Darmspiegelung", die in Deutschland nicht als Kassenleistung angeboten wird.

Der diesem Schreiben beigelegte Flyer zeigt Ihnen die beiden Studienteile und die damit verbundenen Angebote nochmals in der Übersicht. Wir würden uns sehr freuen, wenn Sie sich zu einer Teilnahme entschließen könnten.

Um teilzunehmen, gehen Sie bitte wie folgt vor:

- Bitte lesen Sie vor Ihrer Teilnahme die Teilnehmerinformation sowie die Einverständniserklärung durch.
- Bitte unterschreiben Sie die Einverständniserklärung (1 Exemplar ist für Ihre Unterlagen).
- Bitte füllen Sie den Fragebogen aus.
- Bitte schicken Sie uns die **unterschriebene Einverständniserklärung** und den **ausgefüllten Fragebogen** in dem vorfrankierten Rückumschlag zu.

Bitte beachten Sie, dass die Teilnahme am Studienteil I, d.h. das Ausfüllen des Fragebogens, auch dann möglich und für die Studie sehr hilfreich und wichtig ist, wenn Sie kein Interesse an den Angeboten im Studienteil II haben sollten.

Haben Sie weitere Fragen zu der Studie, können Sie sich natürlich gerne telefonisch (06221 / 56-34322) oder per E-Mail (dario@nct-heidelberg.de) an uns wenden.

Für Ihre Mitwirkung an dieser wichtigen Studie möchten wir uns vorab sehr herzlich bei Ihnen bedanken.

Mit freundlichen Grüßen

Prof. Dr. med. Hermann Brenner
Studienleiter

Petra Schrotz-King

Dr. Petra Schrotz-King
Studienkoordinatorin
