# Supplementary material for: Effects of Alternative Offers of Screening Sigmoidoscopy and Colonoscopy on Utilization and Yield of Endoscopic Screening for Colorectal Neoplasms: Protocol of the DARIO Randomized Trial
Source: JMIR Res Protoc. 2020 Aug 5;9(8):e17516. doi: 10.2196/17516 (PMC7439136; doi:10.2196/17516)
Supplement: Multimedia Appendix 9 [file resprot_v9i8e17516_app9.pdf]

NATIONALES CENTRUM
FÜR TUMORERKRANKUNGEN
HEIDELBERG

getragen von:
Deutsches Krebsforschungszentrum
Universitätsklinikum Heidelberg
Thoraxklinik-Heidelberg
Deutsche Krebshilfe

NCT | Im Neuenheimer Feld 460 (G110) | D-69120 Heidelberg

«P_Anrede»
«AdressName_berechnet»
«P_Strasse_Hausnr» «P_Hausnr» / «P_Adress_Zusatz»
«P_PLZ» «P_Ort»

Prof. Dr. med. Hermann Brenner
Leiter Abteilung Präventive Onkologie

Im Neuenheimer Feld 460
D-69120 Heidelberg

DARIO Studie
Telefon: 06221 56 34322
Telefax: 06221 56-5231
Email: dario@nct-heidelberg.de

Heidelberg, 4. April 2020
Ihr Zeichen: «TN_ID»

DARIO Studie:
Darmkrebsprävention – **I**nn**o**vative Wege am NCT

Einladung zum 2. Teil der DARIO Studie

Sehr geehrte «Ausdr2»,

Herzlichen Dank, dass Sie sich entschieden haben an der DARIO Studie teilzunehmen und uns den Fragenbogen zurückgesendet haben. Damit haben Sie nun die Möglichkeit, auch am Teil II der Studie mitzumachen.

Was bedeutet die weitere Teilnahme für Sie?

Im zweiten Teil der Studie bieten wir Ihnen die Möglichkeit, eine Darmspiegelung bereits vor dem von den gesetzlichen Krankenkassen festgelegten Alter von 55 Jahren durchführen zu lassen.

Wie läuft der zweite Studienteil ab?

Sie wurden von uns bereits nach einem Zufallsverfahren dem Studienarm **A** der DARIO Studie zugewiesen. Als Teilnehmer des Studienarms A können wir Ihnen eine kostenfreie Darmspiegelung (Koloskopie) am Interdisziplinären Endoskopiezentrum (IEZ) des Universitätsklinikums Heidelberg anbieten, eine Untersuchung, die von den Krankenkassen sonst üblicherweise erst ab dem Alter von 55 Jahren angeboten wird.

Rufen Sie uns einfach im DARIO Studienzentrum am Nationalen Centrum für Tumorerkrankungen (NCT) unter der Nummer: 06221 56 34322 an oder schicken Sie uns eine Terminanfrage per Email (dario@nct-heidelberg.de). Gemeinsam buchen wir für Sie einen Termin am IEZ bzw. NCT in Heidelberg zu einem Aufklärungsgespräch für die Darmspiegelung.

Bei Ihrem ersten Termin am IEZ bzw. NCT werden Sie von dem zuständigen DARIO Studienarzt über die Vorgehensweise bei der Darmspiegelung und welche Vorbereitungen Sie dafür treffen müssen, informiert und es wird eine Blutprobe für ein kleines Blutbild entnommen.

Bei diesem Erstgespräch vereinbaren Sie dann auch Ihren 2. Termin, den Koloskopie Termin.

Nach Durchführung der Koloskopie erhalten Sie selbstverständlich das Ergebnis und eine persönliche Empfehlung, wann eine nächste Vorsorgekoloskopie durchgeführt werden sollte.

Nationales Centrum für Tumorerkrankungen (NCT) Heidelberg
Im Neuenheimer Feld 460
D-69120 Heidelberg
www.nct-heidelberg.de

Onkologische Sprechstunden
Allgemeine Onkologie
CUP (Cancer of Unknown Primary)
Dermatologische Tumoren
Gastrointestinale Tumoren
Gynäkologische Tumoren
Gynäkologisch-genetische Sprechstd.
Kopf-Hals Tumoren
Kinderonkologie
Leukämie
Lymphome
Myelome
Neuroendokrine Tumoren
Neuroonkologie
Radioonkologie
Sarkome
Thorakale Tumoren
Urologische Tumoren

Beratungen
Krebsinformationsdienst (KID)
Ernährung
Psychoonkologie
Sozialdienst
Bewegung und Krebs

Geschäftsführende Direktoren
Prof. Dr. Stefan Fröhling (komm.)
Präzisionsonkologie, Deutsches Krebsforschungszentrum (DKFZ)

Prof. Dr. Dirk Jäger
Medizinische Onkologie, Universitätsklinikum Heidelberg (UKHD)

Stellvertretende Direktoren
Prof. Dr. Peter Lichter
Molekulare Genetik, Deutsches Krebsforschungszentrum (DKFZ)

Prof. Dr. Dr. Jürgen Debus
Radioonkologie, Universitätsklinikum Heidelberg (UKHD)

NCT
NATIONALES CENTRUM
FÜR TUMORERKRANKUNGEN
HEIDELBERG

getragen von:
Deutsches Krebsforschungszentrum
Universitätsklinikum Heidelberg
Thoraxklinik-Heidelberg
Deutsche Krebshilfe

Was ist das NCT?

Das Nationale Centrum für Tumorerkrankungen (NCT) Heidelberg ist eine gemeinsame Einrichtung des Deutschen Krebsforschungszentrum (DKFZ), des Universitätsklinikum Heidelberg, der Medizinischen Fakultät Heidelberg und der Deutschen Krebshilfe. Ziel des NCT ist es, vielversprechende Ansätze aus der Krebsforschung möglichst schnell in die Klinik zu übertragen und den Patienten zugutekommen zu lassen. Dies gilt sowohl für die Diagnose, Behandlung, Nachsorge und die Prävention.

Was ist das Interdisziplinare Endoskopie Zentrum?

Am 31.07.06 hat das Interdisziplinäre Endoskopie-Zentrum, kurz IEZ, seinen Betrieb aufgenommen. Im Interdisziplinären Endoskopie Zentrum finden jährlich etwa 10.000 diagnostische und therapeutische endoskopische Untersuchungen statt. Das Zentrum ist personell, räumlich und apparativ auf dem modernsten Stand der Entwicklung. Daher ist eine optimale Behandlung der Patienten, deren Überwachung und die Nachbeobachtung gewährleistet.

Was muss ich tun um mitzumachen?

- Rufen Sie uns an und vereinbaren Sie einen Termin für das Aufklärungsgespräch zur Darmspiegelung unter:

  06221 56 34322 Unsere Sprechzeiten sind:

  Mo - Fr: 10:00-12:00 Uhr zusätzlich **Mi: 13:00-14:30 Uhr**

  Wenn Sie außerhalb unserer Sprechzeiten anrufen, hinterlassen Sie bitte „Ihr Zeichen“ sowie Name, Telefonnummer und Ihr Anliegen auf dem Anrufbeantworter. Wir melden uns dann bei Ihnen.
- Sie kommen zum ärztlichen Aufklärungsgespräch an das IEZ/NCT.
- Sie erhalten ein kleines Dankeschön von uns.
- Den zweiten Termin vereinbaren Sie direkt im IEZ. Das ist der Termin Ihrer Koloskopie.
- Sie erhalten den Befund der Darmspieglung mit einer weiteren persönlichen Empfehlung zur weiteren Darmkrebsvorsorge.

Wir freuen uns darauf Sie bald am IEZ begrüßen zu dürfen. Wann immer Sie weitere Fragen haben zögern Sie nicht, bei uns anzurufen. Gerne können Sie uns auch eine E Mail an Dario@nct-heidelberg.de schicken.

Vielen Dank für Ihre Aufmerksamkeit,
wir freuen uns über Ihre weitere Teilnahme an der Dario Studie

Mit freundlichen Grüßen

Prof. Dr. med. Hermann Brenner Studienleiter

Dr. Petra Schrotz-King Studienkoordinatorin
