# Supplementary material for: Effects of Alternative Offers of Screening Sigmoidoscopy and Colonoscopy on Utilization and Yield of Endoscopic Screening for Colorectal Neoplasms: Protocol of the DARIO Randomized Trial
Source: JMIR Res Protoc. 2020 Aug 5;9(8):e17516. doi: 10.2196/17516 (PMC7439136; doi:10.2196/17516)
Supplement: Multimedia Appendix 11 [file resprot_v9i8e17516_app11.pdf]

NATIONALES CENTRUM FÜR TUMORERKRANKUNGEN HEIDELBERG

getragen von:
Deutsches Krebsforschungszentrum
Universitätsklinikum Heidelberg
Thoraxklinik-Heidelberg
Deutsche Krebshilfe

NCT | Im Neuenheimer Feld 460 (G110) | D-69120 Heidelberg

«P_Anrede»
«AdressName_berechnet»
«P_Strasse_Hausnr» «P_Hausnr» «P_Adress_Zusatz»
«P_PLZ» «P_Ort»

Prof. Dr. med. Hermann Brenner
Leiter Abteilung Präventive Onkologie

Im Neuenheimer Feld 460
D-69120 Heidelberg

DARIO Studie
Telefon: 06221 56 34322
Telefax: 06221 56-5231
Email: dario@nct-heidelberg.de

Heidelberg, 4. April 2020
Ihr Zeichen: «TN_ID»

DARIO Studie:
Darmkrebsprävention – **I**nn**o**vative Wege am NCT

Danke für Ihr Interesse an der DARIO Studie

Sehr geehrter «Ausdr2»,

Herzlichen Dank, dass Sie sich entschieden haben an der DARIO Studie teilzunehmen.

Sie haben durch das Ausfüllen des Fragebogens erfolgreich am Studienteil I teilgenommen. Wir haben anhand Ihrer Angaben geprüft, ob Sie am Studienteil II teilnehmen können.

Sie hatten im Fragebogen Angaben zu früheren Erkrankungen, bzw. Untersuchungen gemacht, die für den Studienteil II als Ausschlusskriterien definiert sind. Das liegt am Aufbau der Studie und ist wissenschaftlich begründet so festgelegt. Wir bedanken uns bei Ihnen sehr, dass Sie bis hierher mitgemacht haben. Sie sind noch immer Teilnehmer am DARIO Studienteil I, auch wenn Sie nun aufgrund der spezifischen Ausschlusskriterien nicht am Studienteil II teilnehmen können. Ihre Angaben sind ein wichtiger und sehr hilfreicher Beitrag zu unserer Studie. Je mehr Personen den Fragebogen ausfüllen, umso fundierter sind in Zukunft unsere Aussagen, wie sich jeder einzelne vor einer Krebserkrankung schützen kann, entweder durch Vorsorgeuntersuchungen oder durch eine Änderung des Lebensstils.

Für Ihr Engagement und für Ihre Teilnahme am Studienteil I der DARIO Studie bedanken wir uns herzlich und wünschen Ihnen für die Zukunft alles Gute.

Mit freundlichen Grüßen

Prof. Dr. med. Hermann Brenner
Studienleiter

Dr. Petra Schrotz-King
Studienkoordinatorin

Nationales Centrum für Tumorerkrankungen (NCT) Heidelberg
Im Neuenheimer Feld 460
D-69120 Heidelberg
www.nct-heidelberg.de

Onkologische Sprechstunden
Allgemeine Onkologie
CUP (Cancer of Unknown Primary)
Dermatologische Tumoren
Gastrointestinale Tumoren
Gynäkologische Tumoren
Gynäkologisch-genetische Sprechstd.
Kopf-Hals Tumoren
Kinderonkologie
Leukämie
Lymphome
Myelome
Neuroendokrine Tumoren
Neuroonkologie
Radioonkologie
Sarkome
Thorakale Tumoren
Urologische Tumoren

Beratungen
Krebsinformationsdienst (KID)
Ernährung
Psychoonkologie
Sozialdienst
Bewegung und Krebs

Geschäftsführende Direktoren
Prof. Dr. Stefan Fröhling (komm.)
Präzisionsonkologie, Deutsches Krebsforschungszentrum (DKFZ)

Prof. Dr. Dirk Jäger
Medizinische Onkologie, Universitätsklinikum Heidelberg (UKHD)

Stellvertretende Direktoren
Prof. Dr. Peter Lichter
Molekulare Genetik, Deutsches Krebsforschungszentrum (DKFZ)

Prof. Dr. Dr. Jürgen Debus
Radioonkologie, Universitätsklinikum Heidelberg (UKHD)
